# Supplementary material for: Epigenetic homogeneity in histone methylation underlies sperm programming for embryonic transcription
Source: Nat Commun. 2020 Jul 13;11:3491. doi: 10.1038/s41467-020-17238-w (PMC7359334; doi:10.1038/s41467-020-17238-w)
Supplement: Supplementary file 1 — Supplementary Information [file 41467_2020_17238_MOESM1_ESM.pdf]

Supplementary Information

“Epigenetic homogeneity in histone methylation underlies sperm programming for embryonic transcription”

Oikawa et al.

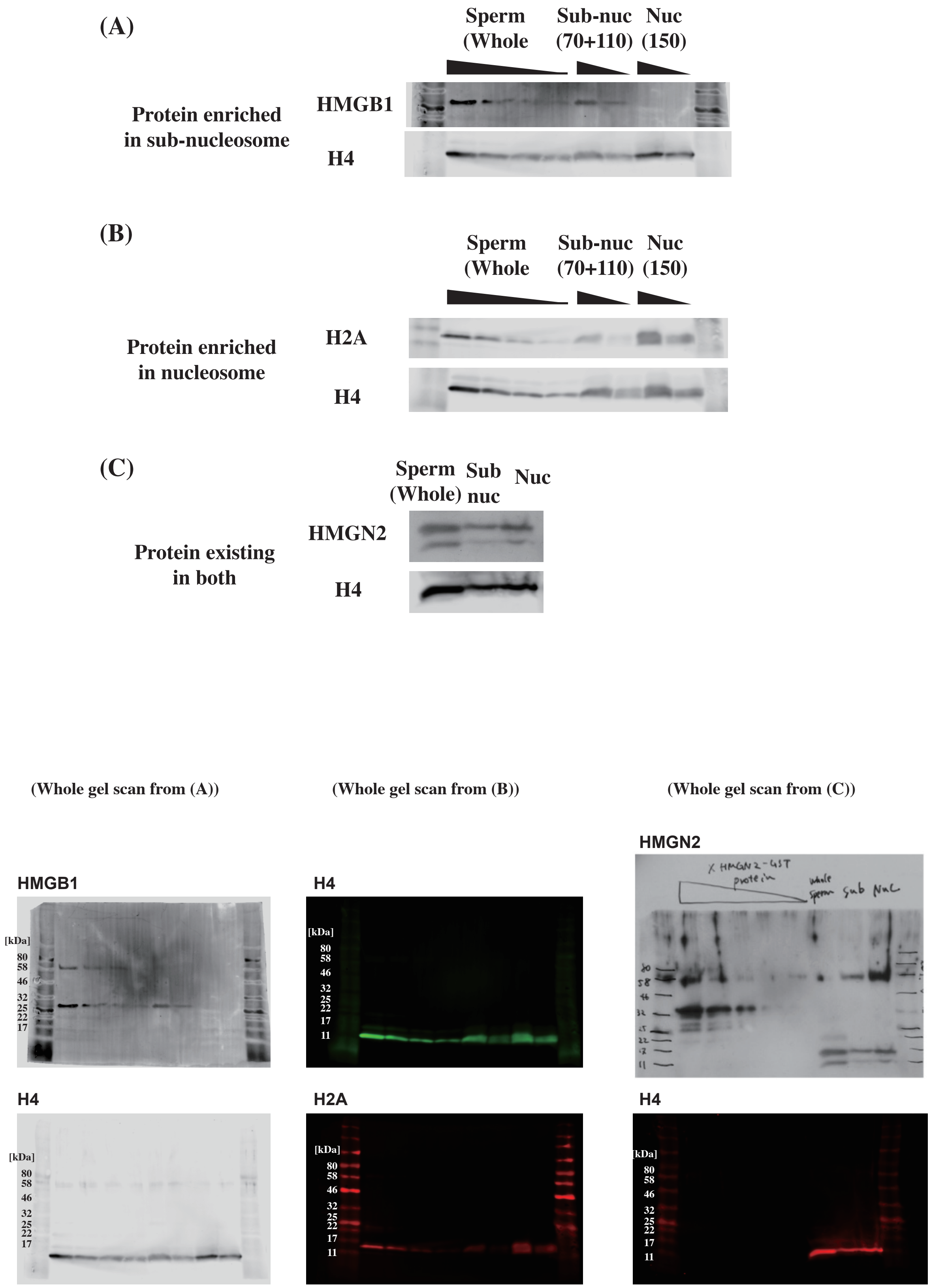
(A)
Sperm (Whole
Sub-nuc (70+110)
Nuc (150)
Protein enriched in sub-nucleosome
HMGB1
H4
(B)
Sperm (Whole
Sub-nuc (70+110)
Nuc (150)
Protein enriched in nucleosome
H2A
H4
(C)
Sperm (Whole)
Sub nuc
Nuc
Protein existing in both
HMGN2
H4
(Whole gel scan from (A))
(Whole gel scan from (B))
(Whole gel scan from (C))
HMGB1
H4
HMGN2
H4
H2A
H4
[kDa]
80
58
46
32
25
22
17
11

FIGURE S1

<u>Supplementary figure 1:</u> ***Proteins associated with nucleosomes and sub-nucleosomes in Xenopus laevis sperm.***

MNase digested sperm chromatin was fractionated by sucrose gradient centrifugation. Nucleosome and subnucleosome fractions were collected and analyzed by Western Blot using antibodies against HMGB1 **(A)**, H2A **(B)**, and HMGN2 **(C)** Whole sperm are used as reference. Anti-histone H4 antibody was used as loading control. WB analysis indicates that HMGB1 is enriched, H2A is depleted, and HMGN2 equally distributed between sub-nucleosome and nucleosome, confirming the results of quantitative mass spectrometry analysis. Each experiment was performed once. Whole gel scan are shown at the bottom. Source data related to panels A, B, and C are provided as Source Data files.

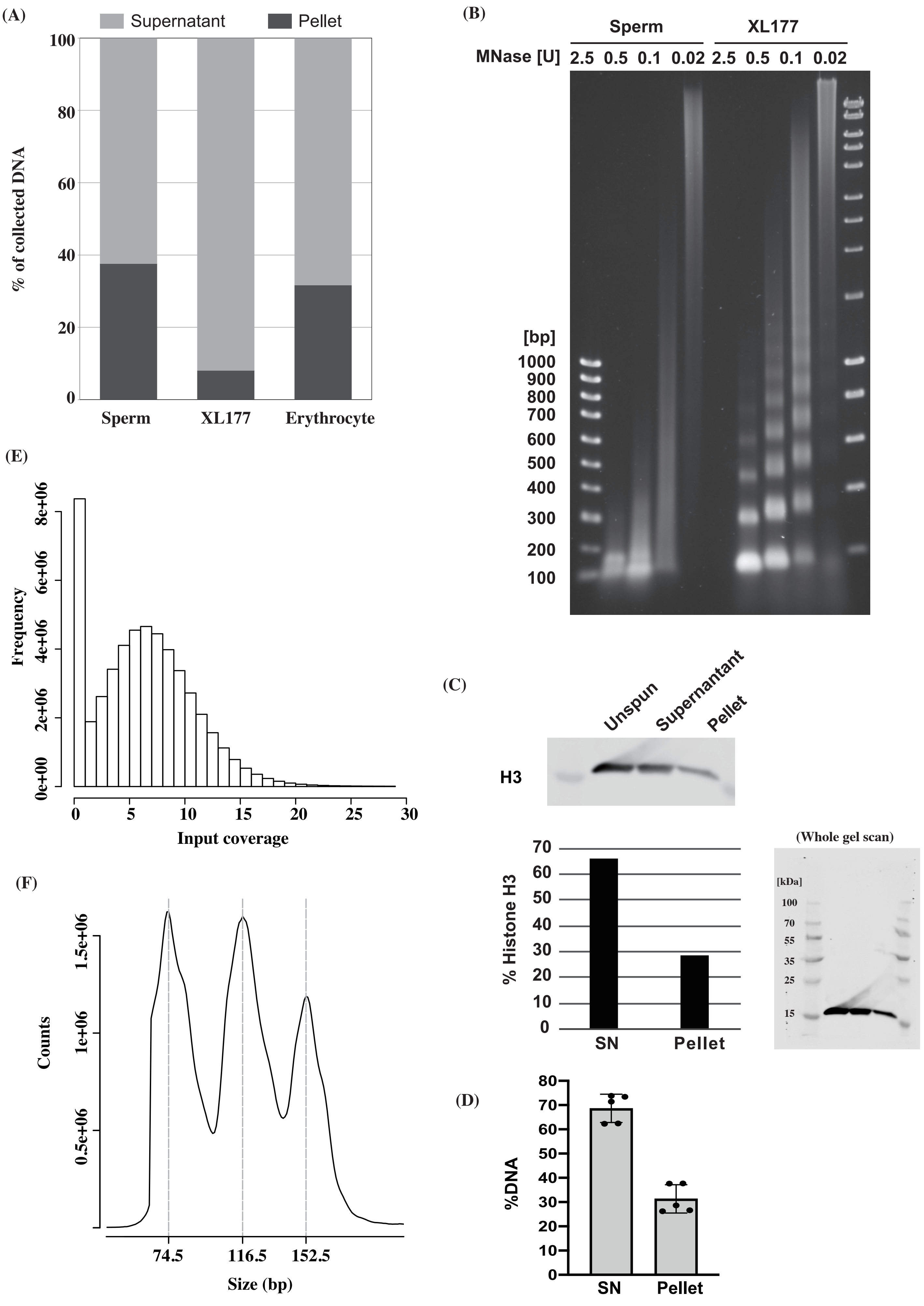

(A)
Supernatant
Pellet
% of collected DNA
100
80
60
40
20
0
Sperm
XL177
Erythrocyte
(B)
Sperm
XL177
MNase [U]
2.5 0.5 0.1 0.02 2.5 0.5 0.1 0.02
[bp]
1000
900
800
700
600
500
400
300
200
100
(E)
Frequency
8e+06
6e+06
4e+06
2e+06
0e+00
0
5
10
15
20
25
30
Input coverage
(C)
Unspun
Supernatant
Pellet
H3
% Histone H3
70
60
50
40
30
20
10
0
SN
Pellet
(Whole gel scan)
[kDa]
100
70
55
35
25
15
(F)
Counts
1.5e+06
1e+06
0.5e+06
74.5
116.5
152.5
Size (bp)
(D)
%DNA
80
70
60
50
40
30
20
10
0
SN
Pellet

FIGURE S2

(G)

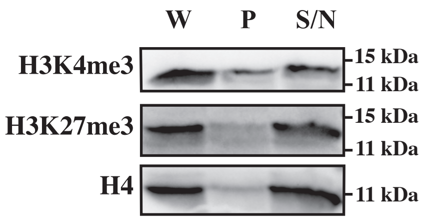

(H)

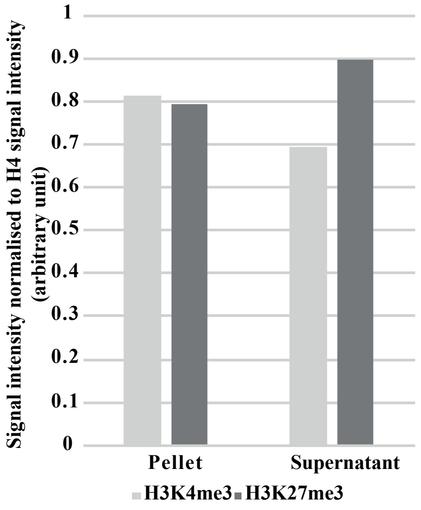

FIGURE S2 (G-H)

Supplementary figure 2: ***Paired-end sequencing of DNA fragments generated by MNase digestion of Xenopus laevis sperm chromatin.***

(A) A large fraction of the sperm genome is solubilized by MNase treatment in sperm. DNA recovery in the supernatant and pellet fraction after 2.5 unit MNase treatment of *Xenopus* sperm or somatic cells (XL177, Erythrocyte). After nuclease digestion, over 60% of the sperm DNA is released as fragments in the 70-200 bp size range, confirming that H3/H4 particles protect a large fraction of the genome, to a similar extent to what is found in the chromatin of a somatic cell (70-90% in XL177 and erythrocyte cells). (n = 1) **(B)** Somatic cell (XL177) and sperm chromatin have a different chromatin structure. 2.5 unit MNase treatment of chromatin generates nucleosome sized fragment in somatic cell and nucleosome plus subnucleosome fragment in sperm (Figure 1B). Chromatin structure difference between somatic cell and sperm is also seen in condition of limited MNase digestion (0.5 to 0.01 unit). A nucleosomal ladder is produced in somatic cell whereas a smear without discrete band is observed in sperm. Nucleosome digestion with 2.5 unit of MNase was repeated in three biological replicates while lower concentration of MNase, 0.5, 0.1, 0.02 unit, were carried out once. **(C)** and **(D)** After digestion of sperm chromatin with 2.5U of MNase, pellets and supernatant were analysed for H3 (C) and DNA (D) content. A similar supernatant/pellet ratio is observed for DNA and histone recovery ((C) n=1 , (D) n=5 , biologically independent samples) **(E)** Histogram of the distribution of input coverage of mapped reads on *X.laevis* genome (pool of input data from two independent replicates). Fragments generated by 2.5 unit MNase treatment of xenopus sperm were paired-end sequenced and mapped onto the *Xenopus* genome. An ~7X average coverage is observed (mean: 6.71, 1stQu:3, 3rdQu:10, median:7). Number of bins in histogram: 30. **(F)** Distribution of paired-end fragment size. The observed local maxima correspond to fragment sizes of: ~70,110 and 150bp. **(G)** After digestion of sperm chromatin with 2.5U of MNase, pellets (P) and supernatant (S/N) were analysed by WB for H4, H3K4me3, and H3K27me3. Undigested sperm (W) is used as control. A representative blot from 4 experiments is shown. **(H)** Ratio of H3K4me3 or H3K4me3 to H4 WB signal intensity in pellets and supernatant fraction from the blot shown in (G). Source data related to panels A,C,D, and G are provided as Source Data files.

(A)

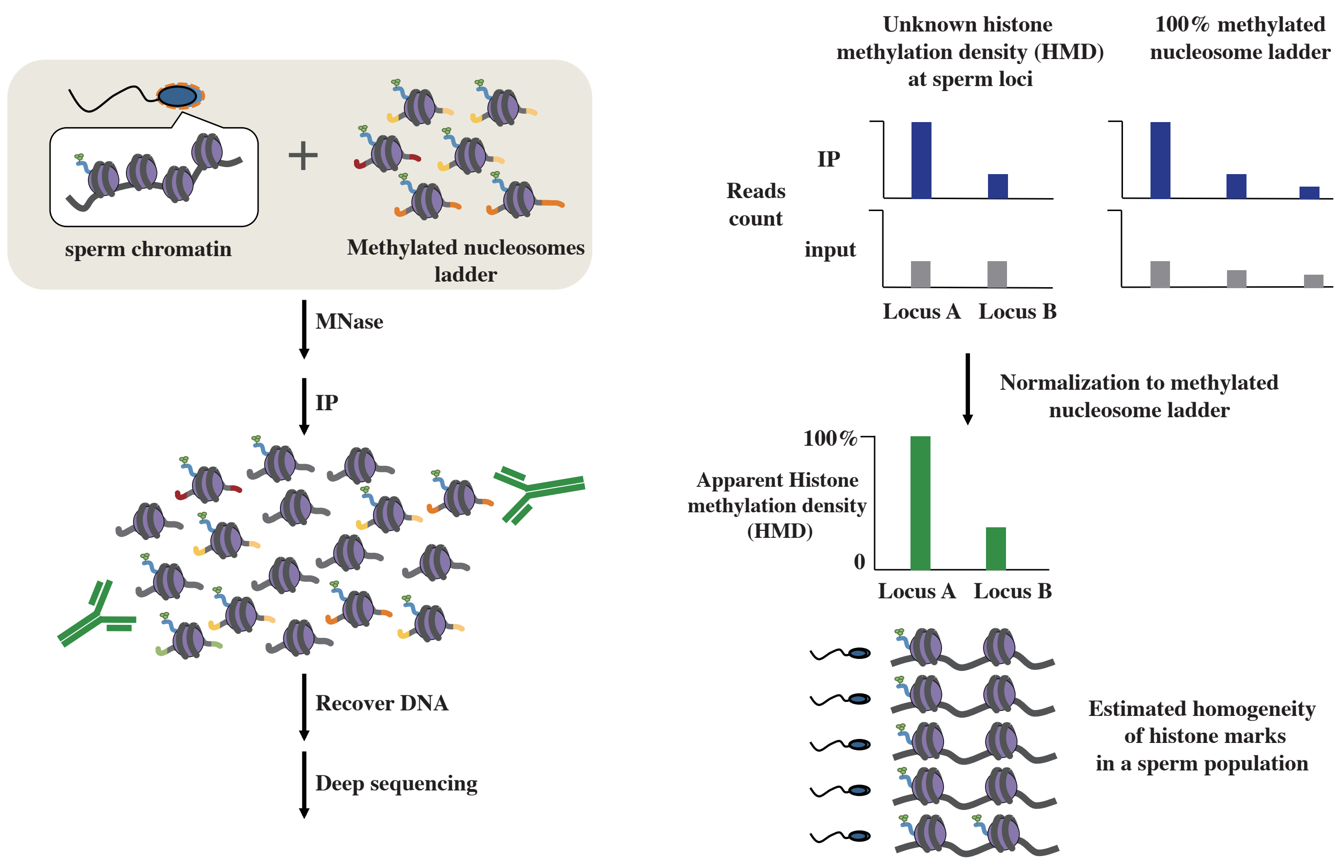

(B)

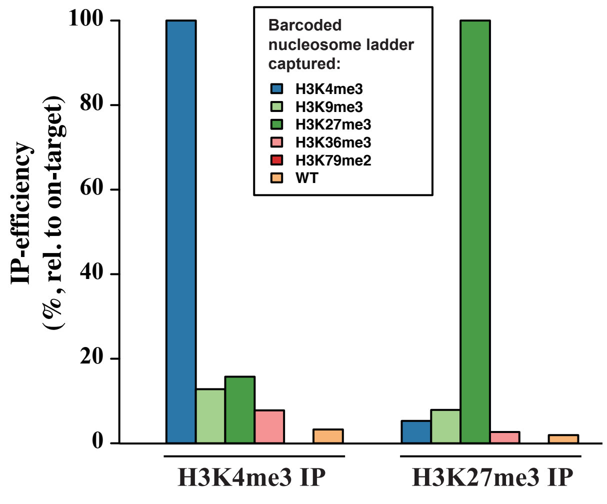

(C)

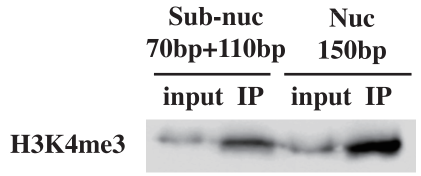

	10% input (Signal intensity)	IP	IP efficiency (%)
Sub-nuc	101000	36600	32.6
Nuc	155000	62800	36.5

(D)

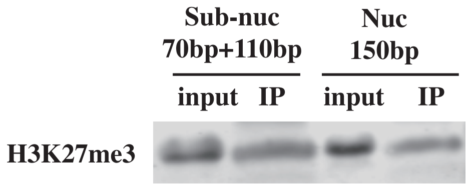

	10% input (Signal intensity)	IP	IP efficiency (%)
Sub-nuc	19800	13700	6.9
Nuc	20500	6940	3.4

FIGURE S3 (A-D)

(E)

Xenopus sperm

	H3K4me3		H3K27me3	
	number of bases	% of bases	number of bases	% of bases
All bases	2.57E+09	100%	2.57E+09	100%
aHMD>80	10923382	0.4%	1.49E+08	5.8%
aHMD>100	6117571	0.2377%	1.13E+08	4.39%
aHMD>150	1588873	0.0617%	54547087	2.12%
aHMD>200	529710	0.0206%	33231879	1.29%
aHMD>300	108939	0.0042%	17242936	0.67%
aHMD>400	35539	0.0014%	10863385	0.42%
aHMD>500	14700	0.0006%	7111516	0.28%
aHMD>900	1550	0.0001%	1494125	0.06%

(F)

mESC

	H3K4me3		H3K27me3	
	number of bases	% of bases	number of bases	% of bases
All bases	2.42E+09	100%	2.42E+09	100%
aHMD>80	2472324	0.1018%	273895918	11.27%
aHMD>100	974506	0.0401%	105612165	4.35%
aHMD>150	213557	0.0088%	23273763	0.95%
aHMD>200	89792	0.0037%	6017672	0.25%
aHMD>300	27783	0.0012%	3110016	0.13%
aHMD>400	10155	0.0004%	835610	0.034%
aHMD>500	4544	0.0002%	263279	0.0108%
aHMD>900	35	0.000001%	24257	0.001%

(G)

Xenopus sperm H3K4me3

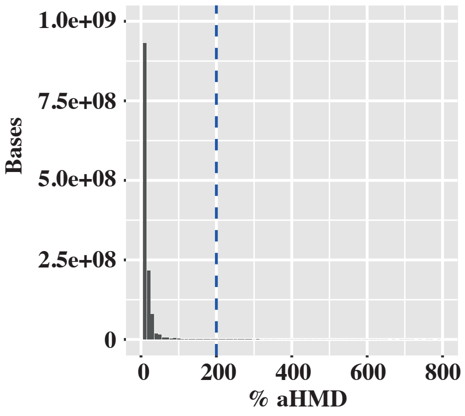

(H)

Xenopus sperm H3K27me3

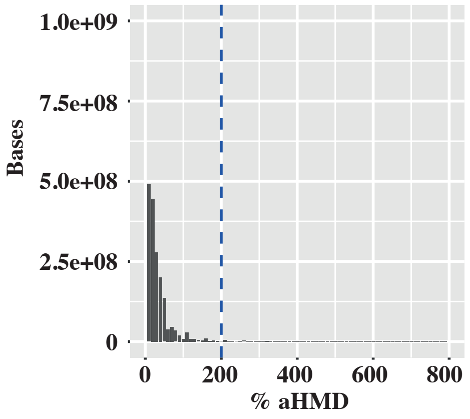

(I)

mESC H3K4me3

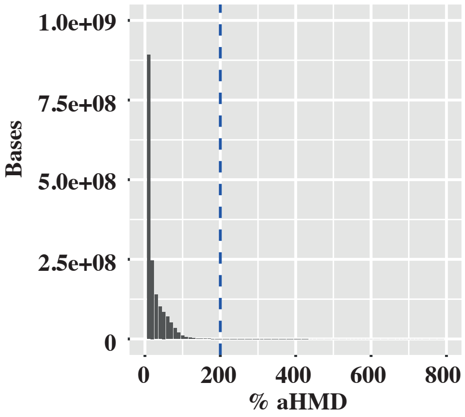

(J)

mESC H3K27me3

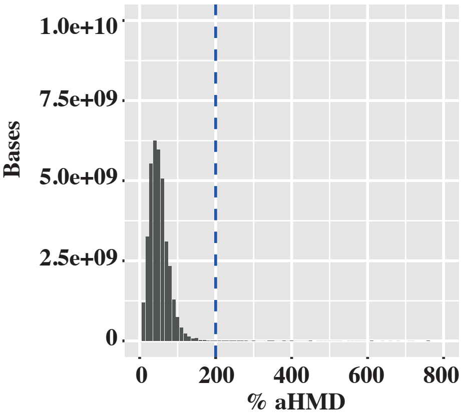

FIGURE S3 (E-J)

<u>Supplementary figure 3:</u> ***Quantitative ChIP-seq analysis (ICe-ChIP) of Xenopus laevis sperm chromatin***

(A) Quantitative ChIP-seq (ICe-ChIP) of *X laevis* sperm was adapted from[2]. Briefly, a nucleosome ladder of fully methylated synthetic nucleosomes bound to barcoded DNAs were added to sperm prior to MNase treatment. Samples were then processed for ChIP-seq analysis. Based on signal obtained from the spiked-in nucleosome ladder, the pull down efficiency at each locus can then be related to an apparent histone methylation density (HMD) at this locus. **(B)** IP specificity: six different barcoded nucleosome ladders (H3K4me3, H3K9me3, H3K27me3, H3K36me3, H3K79me2 and unmodified H3) are simultaneously doped into sperm cell chromatin prior to ICe-ChIP analysis. Pulldown efficiencies for the different nucleosome species are presented as relative IP efficiency, normalized to the cognate target ladder capture (pooled data from two independent replicates). Efficiency of merged pull-down libraries is relative to pulled input libraries and it is computed starting from the actual number of reads mapping to each individual ladder member (for all the different six nucleosomes). Relative efficiency: $(\text{Pull-down efficiency/input efficiency})_{\text{individual\_target}}/\max(\text{Pull-down efficiency/input efficiency})_{\text{tested\_targets}}$, where tested_targets: all individual sequenced spiked-in in the sample. H3K4me3 and H3K27me3 biological replicates: n=2; input libraries: n=2 **(C)** and **(D):** Western blot analysis shows similar efficiency of methylated histone immunoprecipitation from sperm nucleosomes and subnucleosomes. ICe-ChIP quantitation assumes equal IP efficiency of pull down of methylated histone from chromatin to that of the spiked in nucleosome. In the case of *Xenopus* sperm, methylated histones are found both in nucleosomes and subnucleosomes. Nucleosomes and subnucleosomes were purified by sucrose centrifugation of MNase treated sperm chromatin and submitted to immunoprecipitation with an H3K4me3 (C) or H3K27me3 (D) antibody. Starting material as well as the immunoprecipitated fraction were then subjected to quantitative western blot analysis. A similar immunoprecipitation efficiency is observed with nucleosome and subnucleosome. **(E)** and **(F):** The tables report the number and % of bases genome wide corresponding to a given range of apparent Histone Methylation Density (HMD) in *Xenopus* sperm (E) and mouse ESC [2] (F). In both cases occurrences of apparent HMD >100% are restricted to a small fraction of the genome (<0.25% and <4.4% for H3K4me3 and H3K27me3, respectively). **(G)** and **(H)** Frequency of apparent HMD distribution of H3K4me3 (G) and H3K27me3 (F) in *Xenopus* sperm. **(I)** and **(J)** same as G&H for mESC. Source data related to panels C and D are provided as Source Data files.

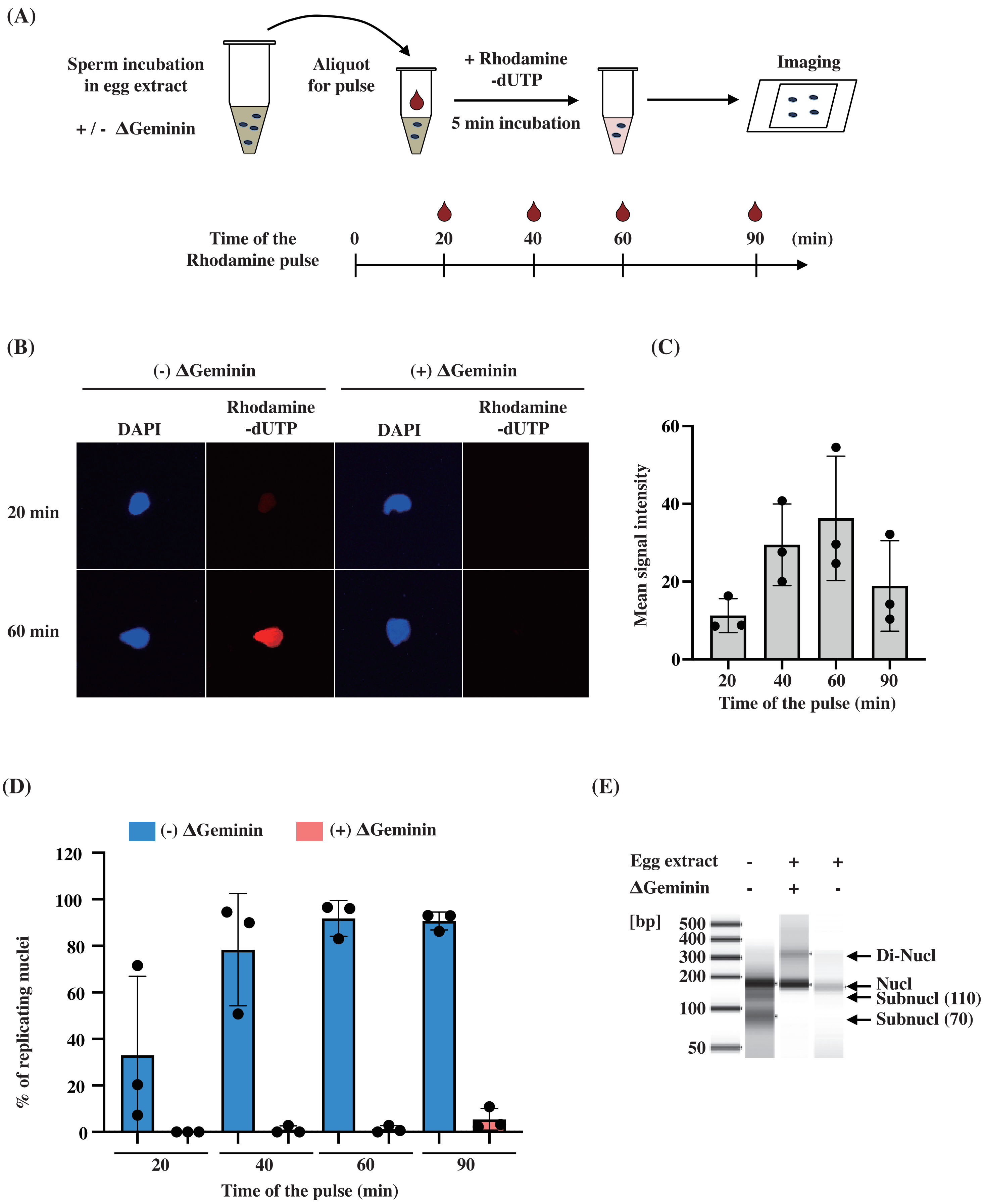
(A)
Sperm incubation in egg extract
+ / - ΔGeminin
Aliquot for pulse
+ Rhodamine -dUTP
5 min incubation
Imaging
Time of the Rhodamine pulse
0
20
40
60
90
(min)
(B)
(-) ΔGeminin
(+) ΔGeminin
DAPI
Rhodamine -dUTP
20 min
60 min
(C)
Mean signal intensity
Time of the pulse (min)
(D)
(-) ΔGeminin
(+) ΔGeminin
% of replicating nuclei
Time of the pulse (min)
(E)
Egg extract
ΔGeminin
[bp]
500
400
300
200
100
50
Di-Nucl
Nucl
Subnucl (110)
Subnucl (70)

FIGURE S4

Supplementary figure 4: ***Chromatin assembly and DNA replication following sperm incubation in egg extract***

(A) Experimental strategy to monitor DNA replication following sperm incubation in egg extract. Rhodamine-dUTP pulse is used to label newly replicated DNA. **(B)** Egg extract mediated replication is inhibited by addition of Δ geminin to the egg extract. Confocal image of sperm 20’ or 60’ following incubation in control or Δ geminin egg extract. **(C)** Sperm DNA replication in extract peaks between 40 -60 min of incubation. Barplot showing quantification of mean rhodamine nuclear intensity in a time course following sperm incubation in extract (n≥30 nuclei/time points, error bar standard deviation from 3 biological replicates) **(D)** Most sperm replicate DNA in egg-extract. Barplot showing the percentage of rhodamine labelled sperm nuclei in sperm incubated into egg extract. (n≥150 nuclei/time points, from 3 biological replicates). Blue bar: incubation with egg only extract; red bar: incubation with egg extract plus geminin **(E)** Chromatin of egg extract treated sperm is remodelled to canonical nucleosomal structure. Tape station analysis of DNA fragment length generated by MNase treatment of sperm before or after extract treatment. Source data related to panels C, D, and E are provided as Source Data files.

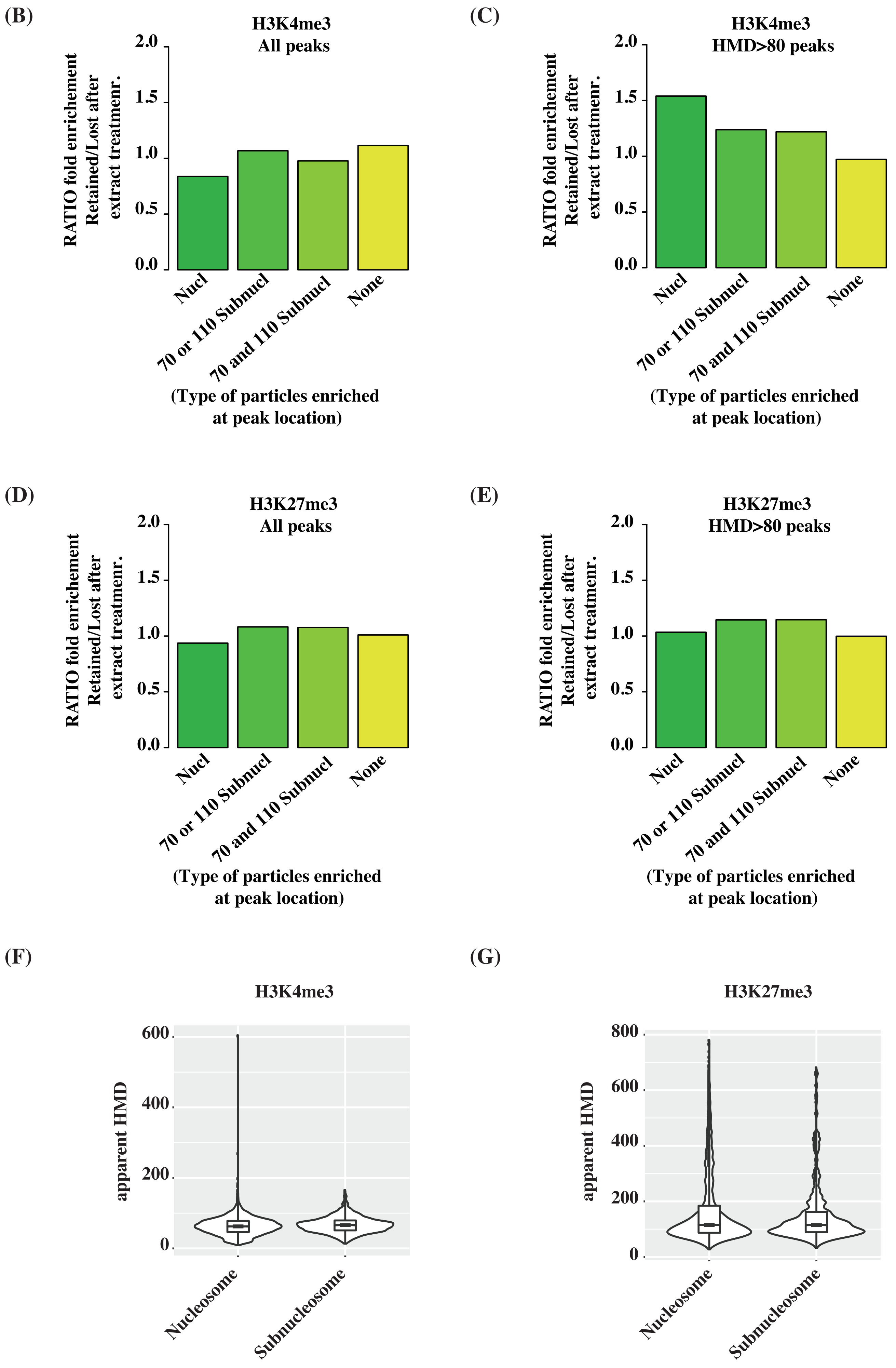
(B)
H3K4me3
All peaks
RATIO fold enrichement
Retained/Lost after
extract treatmenr.
2.0
1.5
1.0
0.5
0.0
Nucl
70 or 110 Subnucl
70 and 110 Subnucl
None
(Type of particles enriched
at peak location)
(C)
H3K4me3
HMD>80 peaks
RATIO fold enrichement
Retained/Lost after
extract treatmenr.
2.0
1.5
1.0
0.5
0.0
Nucl
70 or 110 Subnucl
70 and 110 Subnucl
None
(Type of particles enriched
at peak location)
(D)
H3K27me3
All peaks
RATIO fold enrichement
Retained/Lost after
extract treatmenr.
2.0
1.5
1.0
0.5
0.0
Nucl
70 or 110 Subnucl
70 and 110 Subnucl
None
(Type of particles enriched
at peak location)
(E)
H3K27me3
HMD>80 peaks
RATIO fold enrichement
Retained/Lost after
extract treatmenr.
2.0
1.5
1.0
0.5
0.0
Nucl
70 or 110 Subnucl
70 and 110 Subnucl
None
(Type of particles enriched
at peak location)
(F)
H3K4me3
apparent HMD
600
400
200
0
Nucleosome
Subnucleosome
(G)
H3K27me3
apparent HMD
800
600
400
200
0
Nucleosome
Subnucleosome

FIGURE S5 (B-G)

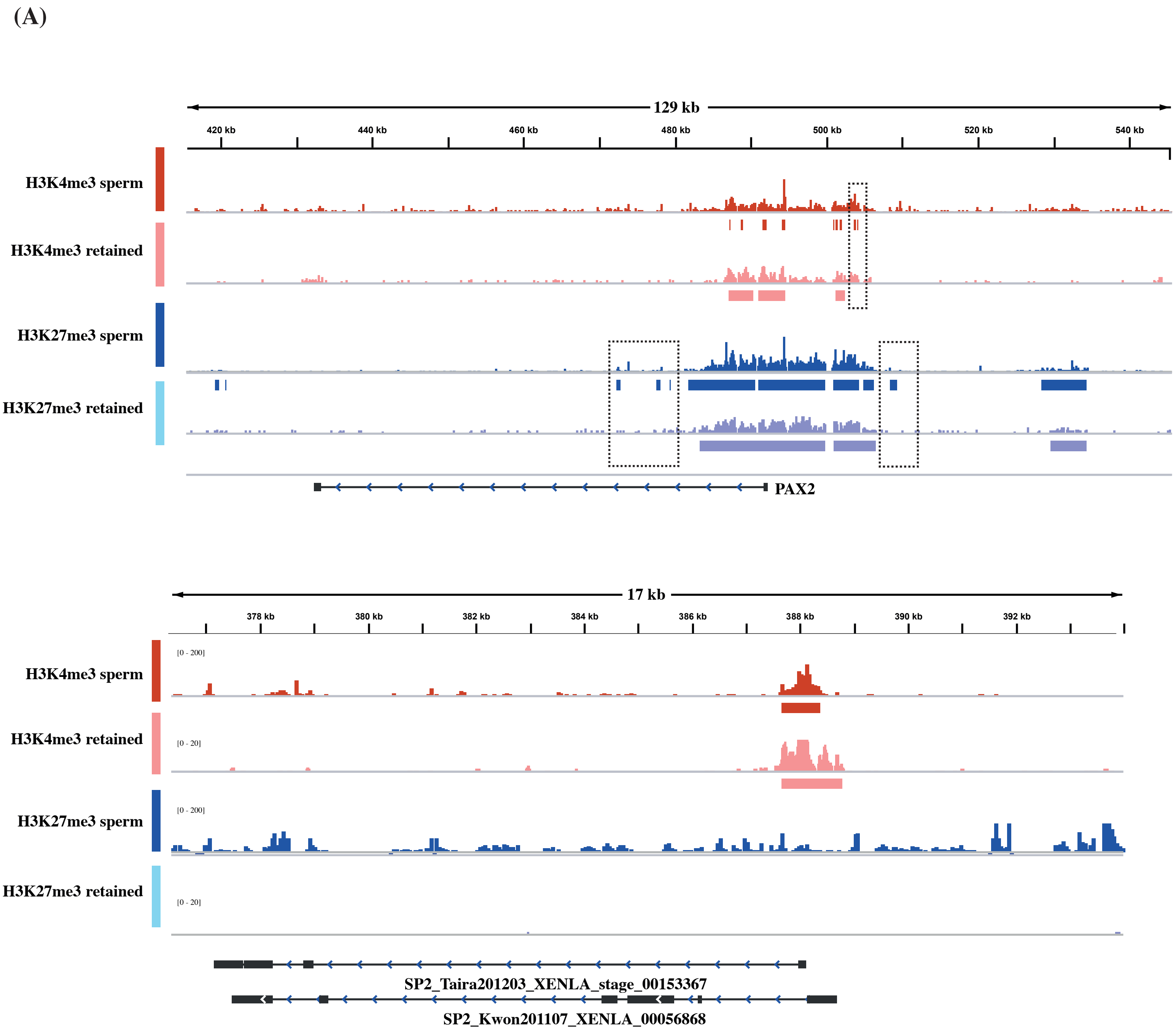

(A)
129 kb
420 kb
440 kb
460 kb
480 kb
500 kb
520 kb
540 kb
H3K4me3 sperm
H3K4me3 retained
H3K27me3 sperm
H3K27me3 retained
PAX2
17 kb
378 kb
380 kb
382 kb
384 kb
386 kb
388 kb
390 kb
392 kb
H3K4me3 sperm
[0 - 200]
H3K4me3 retained
[0 - 20]
H3K27me3 sperm
[0 - 200]
H3K27me3 retained
[0 - 20]
SP2_Taira201203_XENLA_stage_00153367
SP2_Kwon201107_XENLA_00056868

FIGURE S5 (A)

<u>Supplementary figure 5:</u> ***Nucleosome- or subnucleosome- context is generally not predictive of the fate of sperm methylated histones following egg extract treatment***
(A) Browser tracks around Pax2 and SP2 showing H3K4me3 and H3K27me3 signal in sperm and in replicated sperm. Boxes below the track indicate the position of identified peaks. The dashed rectangles highlight regions where peaks are lost after replication in extract. **(B) to (E):** Ratio of fold enrichment at selected genomic location of sperm methylated histone peaks that are retained compared to those that are lost after egg-extract treatment. Selected genomic location are sites with enrichment for : nucleosome; one type of subnucleosome (70 or 110 bp), a mixture of subnucleosomes (70 and 110 bp), or no particular type of particle (none): (B) All sperm H3K4me3 peaks, (C) Sperm H3K4me3 peaks with HMD>80, (D) All sperm H3K27me3 peaks, (E) Sperm H3K27me3 peaks with HMD>80. Ratios are obtained by dividing fold enrichments (observed/random) for each case. 1000 randomizations were carried out and in all instances showed an empirical p-value < 1e-3. **(F)** Boxplots of HMD distribution across H3K4me3 peaks overlapping nucleosomes (n=12296) or subnucleosomes regions (n=5399). **(G)** same as (F) for apparent H3K27 methylation density. Particles enrichment and HMD are from data pooled from two independent replicates. Peaks retention/lost are consensus from 3 independent replicates.

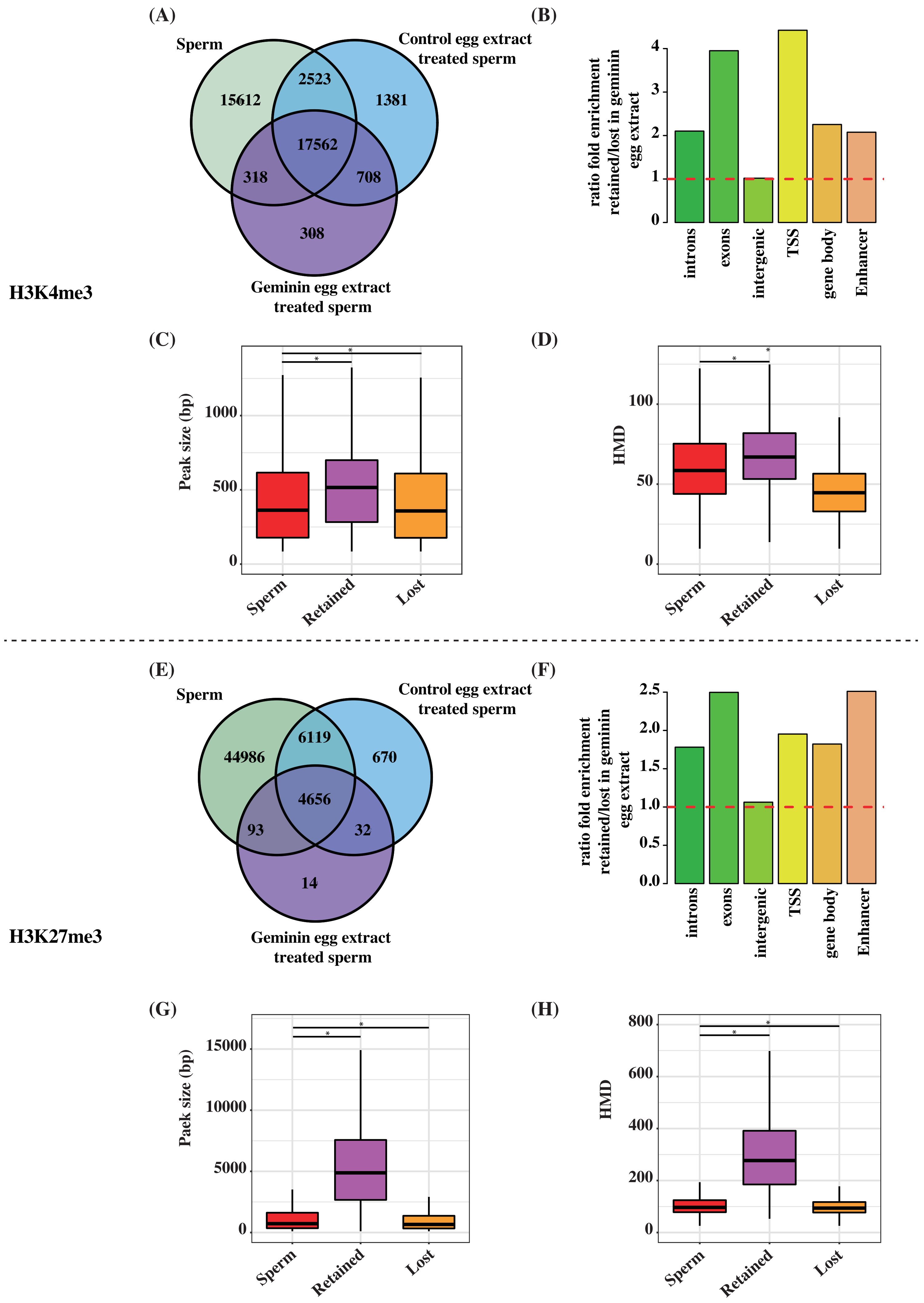

(A)
Sperm
Control egg extract treated sperm
15612
2523
1381
17562
318
708
308
Geminin egg extract treated sperm
H3K4me3
(B)
ratio fold enrichment retained/lost in geminin egg extract
0
1
2
3
4
introns
exons
intergenic
TSS
gene body
Enhancer
(C)
Peak size (bp)
0
500
1000
*
*
Sperm
Retained
Lost
(D)
HMD
0
50
100
*
*
Sperm
Retained
Lost
(E)
Sperm
Control egg extract treated sperm
44986
6119
670
4656
93
32
14
Geminin egg extract treated sperm
H3K27me3
(F)
ratio fold enrichment retained/lost in geminin egg extract
0.0
0.5
1.0
1.5
2.0
2.5
introns
exons
intergenic
TSS
gene body
Enhancer
(G)
Paek size (bp)
0
5000
10000
15000
*
*
Sperm
Retained
Lost
(H)
HMD
0
200
400
600
800
*
*
Sperm
Retained
Lost

FIGURE S6

<u>Supplementary figure 6:</u> ***Fate of sperm methylated histone peaks in replicating and non-replicating egg-extract***

(A)&(E) Venn diagram indicating the overlap between peaks of histone methylation in untreated sperm (green), in sperm treated with replicating extract (control, blue), or treated with non-replicating extract (geminin, purple). **(B)&(F)** ratio of fold enrichment at selected genomic features between peaks that are retained versus lost after geminin extract treatment of sperm. Fold enrichments (observed/random) were obtained over 1000 randomizations and in instances showed an empirical p-value < 1e-3. **(C)&(G)** Boxplots of peaks' size and **(D)&(H)** HMD of all sperm peak (red), peak retained (magenta), and peak lost (orange) after geminin extract treatment. Data in panels **(B) (C) (D)** are obtained using: N. sperm peaks H3K4me3: 36020; N. sperm retained H3K4me3 GMN+: 23156; N sperm lost H3K4me3 GMN+: 12859. Data in panels **(F) (G) (H)** are obtained using: N. sperm peaks H3K27me3: 55854; N. sperm retained H3K27me3 GMN+: 3930; N sperm lost H3K27me3 GMN+: 51919. ***: pvalue < 1e-3 (two-sample Kolmogorov-Smirnov test). HMD are from data pooled from two independent replicates. Peaks retention/lost are consensus from 3 independent replicates.

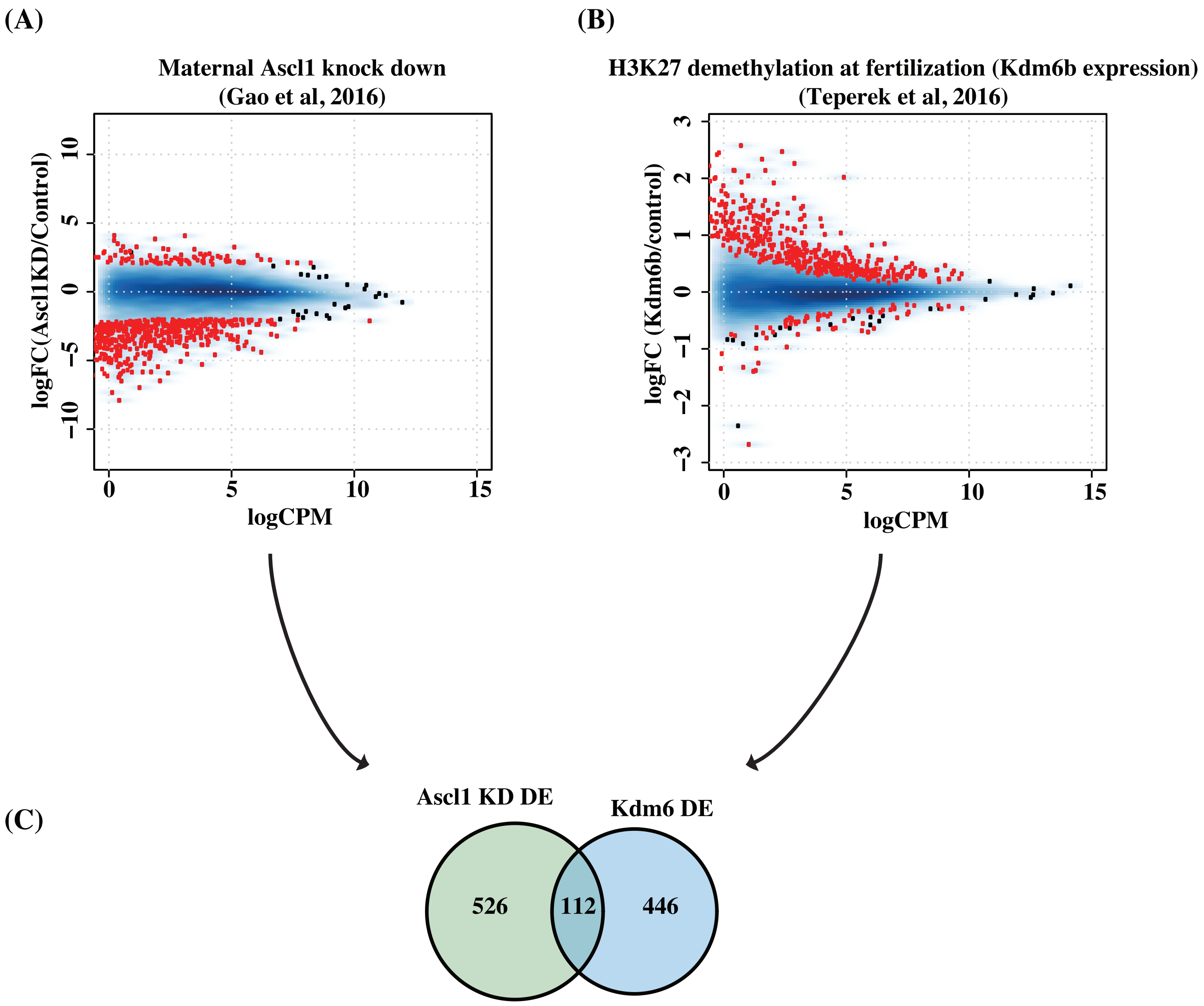

(D)

	Proportion with Ascl 1 motif	Prop.test pval (chi2)	Prop.test pval
(GW) Genes with H3K27me3 HMD>80	12.96%		
(A) Genes DE in AsclKD only and HMD>80	22.62%	41.63	5.514e-11
(B) Genes DE in AsclKD AND Kdm6OE and HMD>80	25.89%	15.38	4.393e-05
(C) Genes DE in Kdm6OE only and HMD>80	39.01%	257	< 2.2e-16

FIGURE S7

<u>Supplementary figure 7:</u> ***Genes with an Ascl1 binding motif homogenously methylated on H3K27 in sperm are misregulated upon either Ascl1 depletion or histone demethylation in early embryos.***

(A) MA plot showing the log fold change (logFC, y-axis) of genes differentially expressed in Ascl1 knock-down versus control (data from [3]). x-axis: expression as log count per million in control embryos; red dot: differentially expressed genes. **(B)** MA plot showing the log fold change (logFC, y-axis) of gene differentially expressed between Kdm6b (H3K27me3 demethylase) injected embryos *versus* control injected embryos. (data from [4]). x-axis: expression as log count per million in control embryos; red dot: differentially expressed genes. **(C)** Venn diagram comparing the sets of differentially expressed genes identified in (A) &(B). **(D)** Gene sets in the three section of the Venn diagram are enriched for genes with an Ascl1 binding sites on the TSS+/-2kB when compared to all sperm genes with high density methylation peaks of H3K27 (HMD>80) on the TSS+/-2kB. Reported p-values corresponds to a proportion test comparing individual case (A, B, or C) to the genome wide proportion (GW).

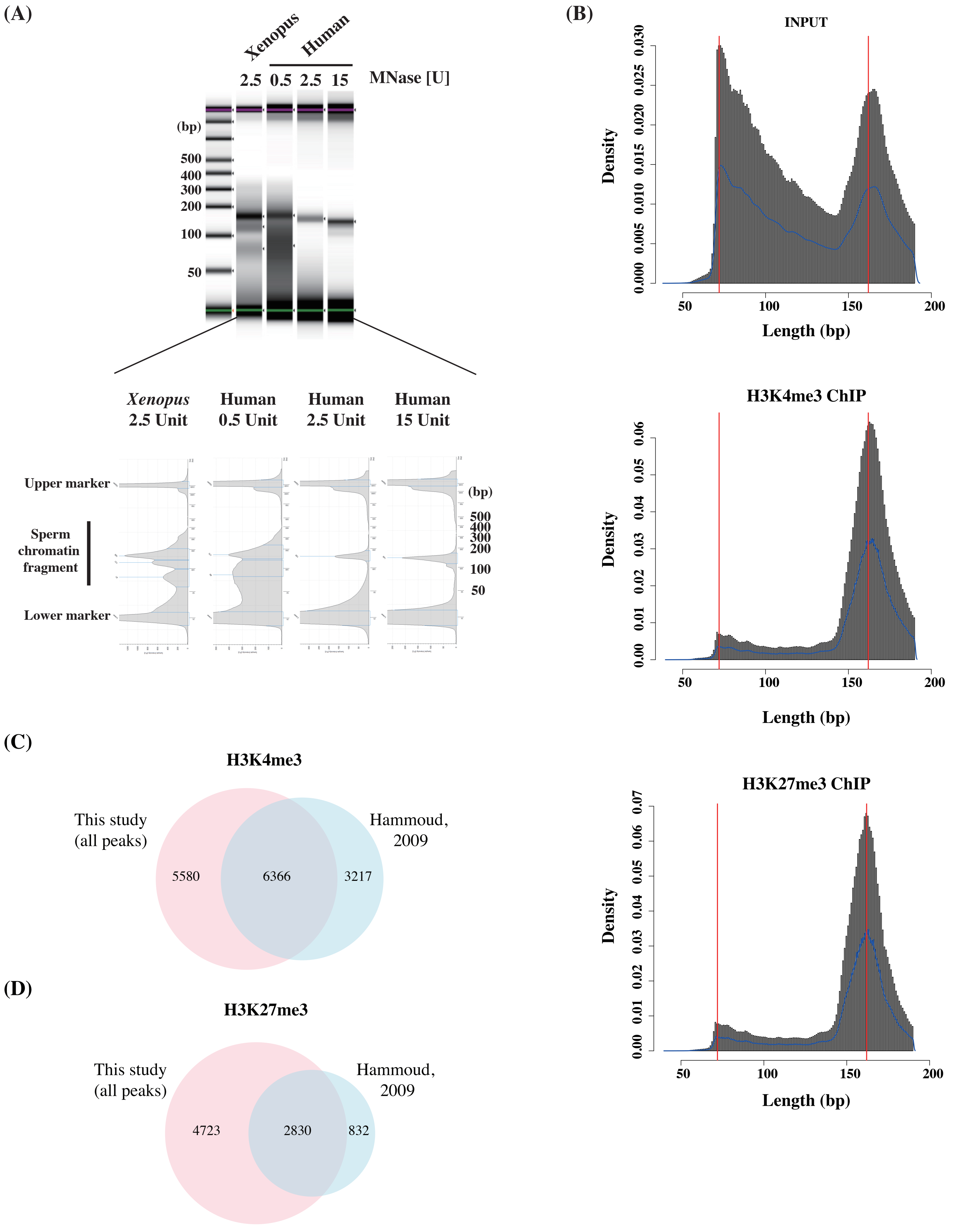

(A)
Xenopus
Human
2.5
0.5
2.5
15
MNase [U]
(bp)
500
400
300
200
100
50
Xenopus 2.5 Unit
Human 0.5 Unit
Human 2.5 Unit
Human 15 Unit
Upper marker
Sperm chromatin fragment
Lower marker
(bp)
500
400
300
200
100
50
(B)
INPUT
Density
Length (bp)
H3K4me3 ChIP
Density
Length (bp)
H3K27me3 ChIP
Density
Length (bp)
(C)
H3K4me3
This study (all peaks)
Hammoud, 2009
5580
6366
3217
(D)
H3K27me3
This study (all peaks)
Hammoud, 2009
4723
2830
832

FIGURE S8

Supplementary figure 8: ***Paired-end sequencing of DNA fragments generated by MNase digestion of human sperm chromatin***

(A) Tape station measurement of DNA fragment sizes obtained by incubation of human sperm with various amount of MNAse. *Xenopus* sperm is used a comparison. Picture of DNA fragments after electrophoresis (top) as well as lane traces (bottom) are shown. **(B)** Histogram of fragments length distribution in Input and H3K4me3 or H3K27me3 ChIP samples. Sperm chromatin was digested with 0.5U of MNase and following IP, Input and ChIP sample were subjected to paired-end sequencing. Fragment length were extracted after mapping paired reads to the genome. **(C)** Overlap of H3K4me3 peaks identified in this study and that of Hammoud *et al.,* 2009[1] . **(D)** Same as (C) for H3K27me3 peaks. Source data related to panel A is provided as Source Data file.

(A)

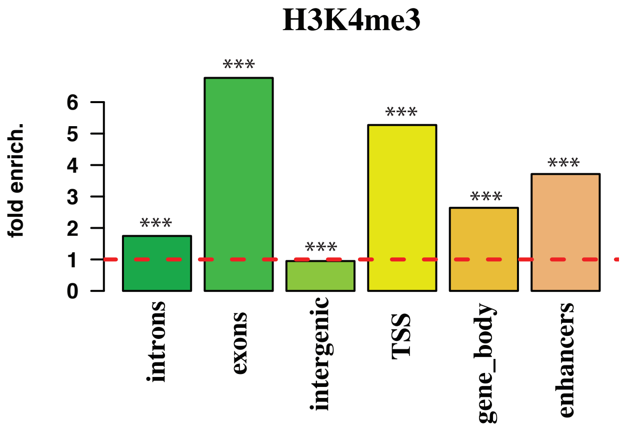
H3K4me3
fold enrich.
0
1
2
3
4
5
6
***
introns
exons
intergenic
TSS
gene_body
enhancers

(B)

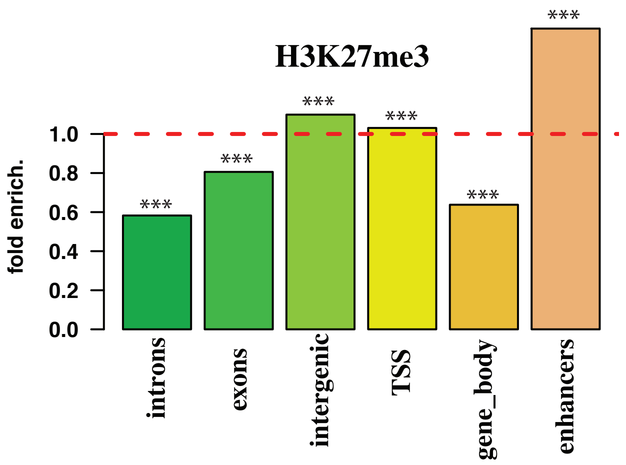
H3K27me3
fold enrich.
0.0
0.2
0.4
0.6
0.8
1.0
***
introns
exons
intergenic
TSS
gene_body
enhancers

FIGURE S9

Supplementary figure 9: ***Peaks of high apparent methylation density are enriched on gene regulatory regions in human sperm.***

Fold enrichment of human sperm peaks with homogeneous histone methylation (HMD >80) at the indicated genomic features for H3K4 **(A)** and H3K27 **(B)**. Fold enrichment (observed/random) are obtained by 1000 randomizations of peaks. All fold enrichments show an empirical p-value < 1e-3. Enhancers are hESC enhancers[5] . Data from one replicate.

Supplementary Methods

Adapter trimming

Adapters have been trimmed using cutadapt (http://dx.doi.org/10.14806/ej.17.1.200). Resulting reads have been aligned to the genome by BWA mem with default options and excluding multiple alignments. The genome used for the alignment is Xlaevis 6.1 for frog and hg38 for human. In the case of IceChIP-seq experiments, a pseudo-genome has been assembled by including the sequence of the synthetic nucleosome ladder [2]. Whenever it was the case, if the same sample was re-sequenced more than one time, bams file with the aligned reads have been merged before identifying duplicates.

Removal of duplicates

Duplicates have been marked with Picard (2.14 - MarkDuplicates) and removed and reads with quality below 20 were also excluded (samtools view –q 20).

Subsequent analysis on paired-end data after alignment

Fragments stratification

BAM files from ICeChIP samples have been sorted, unmapped reads and secondary alignment removed (samtools fixmate). Properly paired reads were joined to obtain fragments size information and stored in BEDPE file format. We extracted BED-format files by considering the beginning and the end of each fragment (i.e. the genomic interval identified by properly paired reads). We used the fragments information to assess the size distribution of fragments used for sequencing.

Based on previous observations and keeping in mind the presence of linker DNA ranging between 20bp-80bp [6-9], we stratified the different classes of fragments on the bases of the size as follows:

- 70bp fragments – containing fragments with size ranging in [60bp,80bp]
- 110bp fragments – containing fragments with size ranging in [100bp,115bp]
- 150bp fragments – containing fragments with size ranging in (115,190]
- 190bp fragments – containing fragment with maximal size of 190

Fragments files 70-110 and 150 were used for the analysis of the chromatin structure organization. This fragments size corresponds also to the local maxima of the fragment size distribution (Figure S2F) and is in line with what observed with western blot of the MNase digested chromatin (Figure 1B).

Files containing fragment 190 were used to estimate levels of histone methylation densities and to call peaks.

Peak calling

Peaks have been called using MACS2 (-q 0.01) using default options for H3K4me3 and –broad option for H3K27me3.

HMD calculation – ladder coverage estimation

For IceChIP-seq data, as indicated in the original work, the coverage of each member of the ladder in both pull down and input was used to estimate the correction factor for each mark. This factor was then used to estimate HMD either genome wide, on the genome binned in 50bp-windows or at detected peaks, as described before [2].

Analysis of input samples for identification of regions enriched for different particle types

We first divided the genome into 50bp windows. For the *Xenopus* data, given that the median number of fragments per window is about or more than 5, we restricted the analysis to those windows where observed 5 or more fragments regardless of their type.
We computed the genome-wide frequency of each of the 3 categories of fragments: f70=0.51, f110=0.14, f150 = 0.36. Given those frequencies, we computed the probability of observing a significant proportion of fragments type in each window for each particle type. We therefore produced a genome-wide p-values' maps.
The recorded p-values have been then discretized into 4 groups:

- GroupNA – if pvalue is NA
- Group0 – if pvalue > 0.1
- Group1 – if pvalue <=0.1 & pvalue > 0.05
- Group2 – if pvalue <=0.05 & pvalue > 0.001
- Group3 – if pvalue <=0.001

We then built a R function that instructs a Hidden Markov Model (HMM) and used it as an unsupervised classificatory system. This function gets as input the discretised probabilities of each fragment type individually and fit an HMM using Baum-Welch algorithm with 2 states. As output the function generated the probability of observing one of the two states. The two states represent enrichment or not enrichment of the particle under investigation. For each particle type, the intervals classified as enriched have been selected for all the subsequent steps of the analysis. In order to parallelise the computation and reduce the computing time we split the probability vector file in multiple parts (bash split), we run the HMM R wrapping function and then we combined the resulting output.
The regions classified as enriched represent sites where the different fragments are more likely to appear compared to the genomic distribution. It is important to note that the regions identified represent genomic locations where a type of particle is likely to be found but it does not imply specific positioning (i.e "phasing") of the particle considered.

ZGA set of genes - alignment of RNA seq and comparison of stg10 to stg8 RNA-Seq data.
RNA-seq data (GSE73430) relative to the embryo at stage 10 and stage 8 have been selected[10]. After adapter trimming (cutadapts, options -q 10 -O 3 –m10), paired-end reads have been aligned to genome version Xlaevis 6.1 using tophat2 (options: -g 1 -p 8 --library-type fr-unstranded). Read counts per transcripts have been extracted using htseq-count (options: -m intersection-strict -s no -t exon -i gene_id). Counts files have been used to perform differential gene expression in R (EdgeR) between stage 10 and stage 8.

Clustering of promoters.
All the different clustering analysis were conducted with a similar approach applied on input data of various kind. We used the partition around medoids (PAM) clustering implemented in the function PAM() from the R package "cluster".
As a common strategy, we looked at the promoters (2 kb around TSS). Those promoter intervals have been binned in 20 windows.
For each of bin we extracted:

1) information about presence/absence of the different type of particle covering it;
2) information about presence of absence of intervals with HMD >= 80;
3) the HMD values.

The 3 types of information have been used alone or in combination for the clustering analysis.

Clustering of promoters based on chromatin particle enrichment.

For this analysis, we considered the binary information about the presence or absence of fragments 150-bp long, interpreted as nucleosomes, or fragments 70+110 bp long representing subnucleosomes and interpreted as a "re-modelled nucleosome".
We binned promoters (20 bins), we computed the occupancy of the two species individually and we codified the information as a 2 digit binary code. The first digit of the code indicated nucleosomes, the second ditig subnucleosome; values 0 and 1 codify absence and presence respectively. Each promoter bins particles structure has been identified as following:

- (1,1): both species occupy the bin
- (0,1): only nucleosomes particle occupies the region
- (1,0): only subnucleosome particle occupy the region:
- (0,0): no particle is detected at the region

The 2-digit binary signature of each bin allowed us to distinguish the type of occupancy occurring. We used it also evaluate Euclidean distance between different promoters where the complete particle vector was obtaining by concatenating all bins of the same promoter together. We performed PAM clustering of the genome-wide promoter chromatin particle vectors using default parameters and we identified 6 clusters.
The results were visualized as a heatmap where 3 colours codify the type of particle occupying each bin and the white indicates the absence of particle. We performed the go analysis (Biological Process BP terms) for the genes in each cluster using the R package TopGO.

Clustering of promoters based on HMD
For clustering the HMD in promoters, we extracted the HMD values of each of the 20 bins per promoter for each histone mark. We combined the two histone marks together and we performed the PAM clustering on the resulting matrix, excluding from the analysis the rows where no signal was detected for the full promoter interval.

Clustering of promoters based on combination of HMD80 and particles type.
For this analysis, we digitalized the information about the presence of HMD>=80 for each individual histone mark and for each of the two categories of particle types (nucleosome, subnucleosomes) in each promoter binned in 20 windows.
This resulted in a footprint of 20 elements for each features of interest: H3K4me3, H3K27me3, nucleosomes (fragments 150), subnucleosomes (fragments 70 and 110bp long). We combined all information into a binary matrix where 1 represent the presence of the feature under consideration and 0 the absence. The matrix comprehended then 80 columns by 34373 genes' promoters. We then performed PAM clustering to identify 6 clusters. We performed the gene enrichment analysis with the R package TopGO.

Transcription factor binding motif enrichment
As input sequences we used the genomic intervals corresponding to identified peaks of homogeneous histone methylation (HMD>80). The search was conducted using default parameters: classic mode, DNA as sequence alphabet, Eukaryote DNA and vertebrate (in vivo and in vitro).

Human data processing
Human data have been processed similarly to the frog's samples. Adapters have been removed (cutadapt) and data were aligned to hg38 genome version with bwa-mem with default options and not allowing for multi-mapping.
Properly-paired reads were used to reconstructs original fragments and saved into BAMBE file format.
As in the frog data, we stratified fragments according to their size.

We computed the coverage of the ladder in both pull-down experiments and input controls and the normalization factors necessary to estimate the histone methylation density (HMD).
HMD has been then estimated for both H3K4me3 and H3K27me3 genome-wide, binning the genome in 50bp consecutive windows and stored in track files (bedgraph, bigwig).
Peaks have been identified by MACS2 (ver 2.1.1, -q 0.01) by using both pull down and input experiments. We identified 11946 peaks for H3K3me3 and 7553 for H3K27me3.
In order to identify genes of interest, we considered those that had a peak satisfying the HMD threshold in their 2kb promoters.
Gene ontology enrichment analysis of genes of interest have been perfomed using DAVID functional enrichment tool (https://david.ncifcrf.gov/)

Human zygotic activation
In order to probe the association with zygotic genome activation, we checked the percentage of ZGA genes [11] with peaks with HMD above threshold. In presence of any overlap we assumed the ZGA gene is positive for the required HMD.

5mC in human sperm
In order to explore the association of HMD and m5C methylation, we considered all 13 samples of bisulphite experiments in human sperm [accession GSE100272]. We binned the genome in windows of 20kb and computed the counts of methylations in each sample. These counts have been normalized by the size of the windows and multiplied by 1000 to have a normalized counts per kilobase in each window. All 13 normalized counts per kilobases have been summed up and then corrected by the density of CpG in the bin. In order to produce the barplot in figure 7E we plotted the HMD values of the peaks and the associated methylation level in the corresponding genomic region.

Conservation between human and frog
In order to look at conservation between frog and human, we run inparanoid [12] using the peptide sequences of each of the two organisms. We processed the output of the orthology mapping performed by inparanoid and we extracted orthologs as pairs of proteins in groups with high-score mapping (score >= 0.95).
We then checked the of genes for which we observed or not a high methylation peak and we assess the proportions of genes conserved between the two organisms.

Supplementary References.

1. Hammoud, S.S. *et al.* Distinctive chromatin in human sperm packages genes for embryo development. *Nature* **460**, 473-478 (2009).
2. Grzybowski, A.T., Chen, Z. & Ruthenburg, A.J. Calibrating ChIP-Seq with Nucleosomal Internal Standards to Measure Histone Modification Density Genome Wide. *Molecular cell* **58**, 886-899 (2015).
3. Gao, L. *et al.* A novel role for Ascl1 in the regulation of mesendoderm formation via HDAC-dependent antagonism of VegT. *Development* **143**, 492-503 (2016).
4. Teperek, M. *et al.* Sperm is epigenetically programmed to regulate gene transcription in embryos. *Genome research* (2016).
5. Tianshun, G. and Jiang, Q. EnhancerAtlas 2.0: an updated resource with enhancer annotation in 586 tissue/cell types across nine species. *Nucleic acids research.*, gkz980, https://doi.org/10.1093/nar/gkz980 (2019).
6. Fei, J. *et al.* The prenucleosome, a stable conformational isomer of the nucleosome. *Genes & development* **29**, 2563-2575 (2015).

7. Luo, D. *et al.* MNase, as a probe to study the sequence-dependent site exposures in the +1 nucleosomes of yeast. *Nucleic acids research* (2018).
8. Rhee, H.S., Bataille, A.R., Zhang, L. & Pugh, B.F. Subnucleosomal structures and nucleosome asymmetry across a genome. *Cell* **159**, 1377-1388 (2014).
9. Rill, R.L. & Nelson, D.A. Histone organization in chromatin: comparison of nucleosomes and subnucleosomal particles from erythrocyte, myeloma, and yeast chromatin. *Cold Spring Harb Symp Quant Biol* **42 Pt 1**, 475-482 (1978).
10. Session, A.M. *et al.* Genome evolution in the allotetraploid frog Xenopus laevis. *Nature* **538**, 336-343 (2016).
11. Li, L. *et al.* Single-cell multi-omics sequencing of human early embryos. *Nat Cell Biol* **20**, 847-858 (2018).
12. Sonnhammer, E.L. & Ostlund, G. InParanoid 8: orthology analysis between 273 proteomes, mostly eukaryotic. *Nucleic acids research* **43**, D234-239 (2015).
