# Supplementary material for: Epigenetic homogeneity in histone methylation underlies sperm programming for embryonic transcription
Source: Nat Commun. 2020 Jul 13;11:3491. doi: 10.1038/s41467-020-17238-w (PMC7359334; doi:10.1038/s41467-020-17238-w)
Supplement: Supplementary file 3 — Description of Additional Supplementary Files [file 41467_2020_17238_MOESM3_ESM.pdf]

Description of Additional Supplementary Files

File Name: Supplementary Data 1
Description: Proteins identified by Mass Spectrometry of purified Xenopus sperm nucleosomal and subnucleosomal particles. The table contains (i) TMT-10 Plex sample Labelling Scheme (ii) Proteome Discoverer 2.1 output result table contains identified and TMT labelled peptide intensities for the Nucleosome and Sub-nucleosome particles at FDR<1% (iii) Normalized peptide intensities of Biological replicates from each condition were compared for the identification of proteins with higher or lower enrichment (log2FC) (iv) Relative abundance of proteins associated with the 150 bp versus 70b bp DNA corresponding to Figure 1 (v) List of proteins identified in the experiment with their complete description.

File Name: Supplementary Data 2
Description: Nucleosomal and subnucleosomal particles enrichment around Xenopus sperm genes. The table contains for each cluster corresponding to figure 2D: (i) the list of genes in that cluster, (ii) the full GO enrichment analysis, and (iii) a filtered GO enrichment analysis (retaining only the GO categories for which a significant p-value is reached in all three statistical tests (classic fischer, Classic KS, and elimKS)).

File Name: Supplementary Data 3
Description: Apparent histone methylation density (HMD) around Xenopus sperm genes. The table contains for each cluster corresponding to figure 3E: (i) the list of genes in that cluster, (ii) the full GO enrichment analysis, and (iii) a filtered GO enrichment analysis (retaining only the GO categories for which a significant p-value is reached in all three statistical tests (classic fischer, Classic KS, and elimKS)). An additional tab “Histone methylation peaks” reports the result of histone methylation peaks analysis using Macs2. Numbers are given corresponding to all H3K4me3 peaks, all H3K27me3 peaks, H3K4me3 only peaks, H3K27me3 only peaks, bivalent peaks (overlap of H3K4 and H3K27 peaks). The analysis is done for peaks regardless of apparent HMD value and for peaks with an apparent HMD value>80%. The tab “genes” reports numbers of genes with TSS=/-2kb associated with peak of H3K4me3 only, H3K27me3 only, or both (“bivalent”)..

File Name: Supplementary Data 4
Description: Combined analysis of particles enrichment and apparent histone methylation density (HMD) around Xenopus sperm genes.The table contains:
- Fold enrichments and corresponding statistical significance (empirical p-value obtained after performing 1000 randomizations) for fold enrichments reported in fig 2C, fig 3C, fig 4A-B, fig 5E, fig 5I, sup. fig 5 B-E, sup. fig 6B, sup. fig 6E.
- HMD distribution ranges of boxplots in fig. 5C-D, fig 5 G-H and the outcome of the statistical test performed to cross compare them. Differences have been tested with Chi2 two-sample proportion test, one-sided.
- For each cluster corresponding to figure 4C: (i) the list of genes in that cluster, (ii) the full GO enrichment analysis, and (iii) a filtered GO enrichment analysis (retaining only the GO categories for which a significant p-value is reached in all three statistical tests (classic Fisher, Classic KS, and elimKS)).

File Name: Supplementary Data 5
Description: Genes sets related to Figure 6. Lists of genes corresponding to all the gene sets considered in figure 6.

File Name: Supplementary Data 6
Description: Apparent histone methylation density (HMD) around human sperm genes.
The table contains for each cluster corresponding to figure 7B: (i) the list of genes in that cluster, (ii) the full GO enrichment analysis, and (iii) a filtered GO enrichment analysis (retaining only the GO categories for which a significant p-value is reached in all three statistical tests (classic Fisher, Classic KS, and elimKS)).

File Name: Supplementary Data 7
Description: Histone methylation peaks in human sperm genes
The tab “Hammoud,2009 & this study” indicates the number of genes with a peak of H3K4me3 or H3K27me3 peaks in human sperm for this study , for that of Hammoud et al.1, and the overlap between the two studies. The tab “Comparisons_Human & Xenopus” indicates the number of orthologs marked by H3K4me3 or H3K27me3 in Xenopus and human sperm, stratified by apparent HMD level.

File Name: Supplementary Data 8
Description: Genes with high apparent histone methylation density (HMD) in human sperm.
GO analysis of human sperm genes associated with a peak of apparent HMD >80% for H3K4me3 or for H3K27me. Gene ontology enrichment analysis of genes of interest have been perfomed using DAVID functional enrichment tool (https://david.ncifcrf.gov/). the p-value are from the modified fisher exact test.

File Name: Supplementary Data 9
Description: FASTA file listing all xenopus transcripts used in this analysis.
