# Supplementary material for: Epigenetic homogeneity in histone methylation underlies sperm programming for embryonic transcription
Source: Nat Commun. 2020 Jul 13;11:3491. doi: 10.1038/s41467-020-17238-w (PMC7359334; doi:10.1038/s41467-020-17238-w)
Supplement: Supplementary file 13 — Reporting Summary [file 41467_2020_17238_MOESM13_ESM.pdf]

nature research

Corresponding author(s): Jerome Jullien

Last updated by author(s): 09/03/2020

Reporting Summary

Nature Research wishes to improve the reproducibility of the work that we publish. This form provides structure for consistency and transparency in reporting. For further information on Nature Research policies, see Authors & Referees and the Editorial Policy Checklist .

Statistics

For all statistical analyses, confirm that the following items are present in the figure legend, table legend, main text, or Methods section.

n/a	Confirmed	
☐	☒	The exact sample size (*n*) for each experimental group/condition, given as a discrete number and unit of measurement
☐	☒	A statement on whether measurements were taken from distinct samples or whether the same sample was measured repeatedly
☐	☒	The statistical test(s) used AND whether they are one- or two-sided<br>*Only common tests should be described solely by name; describe more complex techniques in the Methods section.*
☐	☒	A description of all covariates tested
☐	☒	A description of any assumptions or corrections, such as tests of normality and adjustment for multiple comparisons
☐	☒	A full description of the statistical parameters including central tendency (e.g. means) or other basic estimates (e.g. regression coefficient) AND variation (e.g. standard deviation) or associated estimates of uncertainty (e.g. confidence intervals)
☐	☒	For null hypothesis testing, the test statistic (e.g. *F*, *t*, *r*) with confidence intervals, effect sizes, degrees of freedom and *P* value noted<br>*Give P values as exact values whenever suitable.*
☒	☐	For Bayesian analysis, information on the choice of priors and Markov chain Monte Carlo settings
☐	☒	For hierarchical and complex designs, identification of the appropriate level for tests and full reporting of outcomes
☐	☒	Estimates of effect sizes (e.g. Cohen's *d*, Pearson's *r*), indicating how they were calculated

Our web collection on statistics for biologists contains articles on many of the points above.

Software and code

Policy information about availability of computer code

Data collection	No computer code were used to collect the data.

Data analysis

ChIP-seq and RNA-seq analysis: Adapters have been trimmed using cutadapt (v1.0). Resulting reads have been aligned to the genome by BWA (v0.6.2) mem with default options and excluding multiple alignments. The genome used for the alignment is Xlaevis 6.1 for frog and hg38 for human. Duplicates have been marked with Picard (v2.14 - MarkDuplicates) and removed and reads with quality below 20 were also excluded (samtools view –q 20) with samtools (v0.1.8). BAM files from ICeChIP samples have been sorted, unmapped reads and secondary alignment removed (samtools fixmate). Properly paired reads were joined to obtain fragments size information and stored in BEDPE file format with bedtools (v2.25.0). Peaks have been called using MACS2 (v2.1.1, -q 0.01) using default options for H3K4me3 and broad option for H3K27me3. For analysis of ICeChIP-seq data, as indicated in the original work, the coverage of each member of the ladder in both pull down and input was used to estimate the correction factor for each mark. This factor was then used to estimate HMD either genome wide, on the genome binned in 50bp-windows or at detected peaks, as described before (Grzybowski et al., 2015). For analysis of input samples for identification of regions enriched for different particle types, we built a R (v3.2.4) function that instructs a Hidden markov Model (HMM) and used it as an unsupervised classificatory system. For analysis of published RNA-seq data (GSE73430), we performed adapter trimming (cutadapts, options -q 10 -O 3 –m10). paired-end reads have been aligned to genome version Xlaevis 6.1 using tophat2 (options: -g 1 -p 8 --library type fr-unstranded). Read counts per transcripts have been extracted using htseq-count (options: -m intersection-strict -s no -t exon -i gene_id). Counts files have been used to perform differential gene expression in R (EdgeR).

Image analysis: Signal intensity of images were analyzed with Image J (http://imagej.nih.gov/ij/)

Quantitative Mass spectrometry data processing: The collected CID (collision induced dissociation) tandem mass spectra were processed with the SequestHT search engine against the Xenopus laevis proteome database on the Proteome Discoverer 2.1 software for peptide and protein identifications. Quantitative proteomics data were analysed by qPLEXanalyzer, an R Bioconductor package.

For manuscripts utilizing custom algorithms or software that are central to the research but not yet described in published literature, software must be made available to editors/reviewers. We strongly encourage code deposition in a community repository (e.g. GitHub). See the Nature Research guidelines for submitting code & software for further information.

Data

Policy information about availability of data

All manuscripts must include a data availability statement. This statement should provide the following information, where applicable:
- Accession codes, unique identifiers, or web links for publicly available datasets
- A list of figures that have associated raw data
- A description of any restrictions on data availability

We will deposit all the ChIP-seq and ICeChIP-seq data generated in this study into the Gene Expression Omnibus and will inform their accession number as soon as possible.
Xenopus laevis genome data (JGI version 6.1) were from Xenopus genome project (ftp://ftp.xenbase.org/pub/Genomics /JGI/Xenla6.1/). Mouse ES cell ICeChIP-seq data were downloaded from GSE60378. Stage 10 to stage 8 Xenopus embryo RNA-seq data were downloaded from GSE73430. KDM5B or KDM6B injected stage 10.5 Xenopus embryo RNA-seq data were downloaded from GSE75164. Control or maternal Ascl1 knock down, stage 10.5 Xenopus embryo RNA-seq data were downloaded from GSE76915. Human sperm ChIP-seq data were downloaded from GSE15594. Human preimplantation embryo RNA-seq data were downloaded from CRA000297. Human sperm scCOOL-seq were downloaded from GSE100272.

Field-specific reporting

Please select the one below that is the best fit for your research. If you are not sure, read the appropriate sections before making your selection.

- [x] Life sciences
- [ ] Behavioural & social sciences
- [ ] Ecological, evolutionary & environmental sciences

For a reference copy of the document with all sections, see nature.com/documents/nr-reporting-summary-flat.pdf

Life sciences study design

All studies must disclose on these points even when the disclosure is negative.

Sample size	1 x 10*6 of Xenopus laevis sperm, 0.5 x 10*6 of egg extract treated Xenopus laevis sperm, 150 of St7 Xenoous embryos, 50 x 10*6 of human sperm per reaction for ChIP-seq.
Data exclusions	No data were excluded from the analyses.
Replication	ChIP-seq were performed for each antibody and samples with 2-3 of replications.
Randomization	All sperm were collected from the testis from male Xenopus laevis or men. Immunoprecipiated sperm chromatin were randomly allocated for ChIP-seq.
Blinding	This study does not include experiments which is needed to consider the influences from testers's preferences or expectations.

Reporting for specific materials, systems and methods

We require information from authors about some types of materials, experimental systems and methods used in many studies. Here, indicate whether each material, system or method listed is relevant to your study. If you are not sure if a list item applies to your research, read the appropriate section before selecting a response.

Materials & experimental systems

n/a	Involved in the study
☐	☒ Antibodies
☐	☒ Eukaryotic cell lines
☒	☐ Palaeontology
☐	☒ Animals and other organisms
☐	☒ Human research participants
☒	☐ Clinical data

Methods

n/a	Involved in the study
☐	☒ ChIP-seq
☒	☐ Flow cytometry
☒	☐ MRI-based neuroimaging

Antibodies

Antibodies used

Histone H3K4me3 primary antibody (Abcam, ab8580), 2 ug per ChIP reaction, 1/1000 dilution for western blotting
Histone H3K27me3 primary antibody (king gift from Dr. Thomas Jenuwein), 2 ul per ChIP reaction, 1/1000 dilution for western blotting
Histone H4 primary antibody (Abcam, ab31830), 1/1000 dilution for western blotting
Histone H3 primary antibody (Cell signaling, #14269), 1/1000 dilution for western blotting
Histone H2A primary antibody (Millipore, 07-146), 1/1000 dilution for western blotting
Histone H2B primary antibody (Abcam, ab1790), 1/1000 dilution for western blotting
HMGB1 primary antibody (Sigma, H9664), 1/1000 dilution for western blotting
HMGN2 primary antibody (kind gift from Dr. Hock), 1/1000 dilution for western blotting
Alexa 680 secondary antibody (Thermo Fisher, A21058), 1/25000 dilution for western blotting
Alexa 800 secondary antibody (Thermo Fisher, A32735), 1/25000 dilution for western blotting
Goat anti-Rabbit IgG (H+L) Secondary Antibody, HRP (Thermo Fisher, #31460), 1/10000 dilution for western blotting

Validation

Histone H3K4me3 antibody (abcam, ab8580): This antibody can be used ChIP, immunoprecipitation, Flow cytometry, Western Blotting and Immunofluorescence in Mouse, Rat, Rabbit, Human, Pig, Saccharomyces cerevisiae, Tetrahymena, Xenopus laevis, Arabidopsis thaliana, Caenorhabditis elegans, Drosophila melanogaster, Zebrafish, Trypanosoma cruzi, Common marmoset, Rice, Xenopus tropicalis according to the manufacture's description. There are validation data for ChIP with U-2 OS cells on the manufacture's website. In our experiment, we validated this antibody by performing ChIP-seq with Xenopus and human sperm, and WB with Xenopus sperm.

Histone H3K27me3 antibody (generous gift from Dr. Thomas Jenuwein): This antibody can be used ChIP, Western Blotting in Mouse, Xenopus laevis according to his group (Peters et al., 2003) and our previous results (Teperek et al., 2016). In our experiment, we validated this antibody by performing ChIP-seq with Xenopus and human sperm.

Histone H4 antibody (abcam, ab31830): This antibody can be used ChIP, immunoprecipitation, Flow cytometry, Western Blotting and Immunofluorescence in Cow and Human, predicted to work with Mouse and Rat according to the manufacture's description. There are validation data for WB with Calf Thymus or HeLa Histone preparation nuclear lysate on the manufacture's website. In our experiment, we validated this antibody by performing WB with Xenopus sperm.

Histone H3 antibody (Cell signaling, #14269): This antibody can be used ChIP, immunoprecipitation, Flow cytometry, Western Blotting and Immunofluorescence in Human, Mouse, Rat, Monkey according to the manufacture's description. There are validation data for WB with HeLa, NIH/3T3, C6, COS-7 cell extracts on the manufacture's website. In our experiment, we validated this antibody by performing WB with Xenopus sperm.

Histone H2B antibody (Abcam, ab1790): This antibody can be used ChIP and Western Blotting, immunoprecipitation and Immunofluorescence in Mouse, Rat, Human, Chicken, Cow, Saccharomyces cerevisiae, Xenopus laevis, and Arabidopsis thaliana, Zebrafish according to the manufacture's description. There are validation data for WB with HeLa cell lysate on the manufacture's website. In our experiment, we validated this antibody by performing WB with Xenopus sperm.

HMGB1 antibody (Sigma, H9664): This antibody can be used Western Blotting, immunoprecipitation and Immunofluorescence in Mouse, Rat and Human according to the manufacture's description. There are validation data for WB with HeLa, NIH3T3 and HEK-293T cell lysate on the manufacture's website. In our experiment, we validated this antibody by performing WB with Xenopus sperm.

HMGN2 antibody (generous gift from Dr. Robert Hock): This antibody can be used Western Blotting in Xenopus laevis according to his group (Körner et al., 2003). In our experiment, we validated this antibody by performing WB with Xenopus sperm.

Eukaryotic cell lines

Policy information about cell lines

Cell line source(s)	The XL177 cell line was a kind gift from Dr. James C. Smith (Smith et al., 1987). It was established from Xenopus laevis embryos and used in several papers (Collart et el., 2011, Teperek et al., 2016).
Authentication	No authentication.
Mycoplasma contamination	Not tested.
Commonly misidentified lines (See ICLAC register)	No commonly misidentified cell lines were used.

Animals and other organisms

Policy information about studies involving animals; ARRIVE guidelines recommended for reporting animal research

Laboratory animals	Mature Xenopus laevis laevis male (Nasco, LM00715MX) and female (Nasco, LM00535MX) were used in this study.
Wild animals	No wild animals were used in this research.
Field-collected samples	No field-collected samples were used in this research.
Ethics oversight	Our work with Xenopus laevis is covered under the Home Office Project License PPL 70/8591 and frog husbandry and all experiments were performed according to the relevant regulatory standards. The researchers and the staff of the Gurdon Institute animal husbandry facility are trained in these experiments, and veterinarians monitor the health status of the animals.

Note that full information on the approval of the study protocol must also be provided in the manuscript.

Human research participants

Policy information about studies involving human research participants

Population characteristics	Human sperm donors for experiments were anonymous. Men were deemed fertile based on the following parameters; female partners are currently pregnant or have given birth within 6 months of sample production. Semen samples were collected from men aged between 30-35 years old.
Recruitment	Participants are recruited at ReproMed Ireland, Rockfield Medical Campus, Northblock, Dundrum, Dublin 16, D16 20 W7W3.
Ethics oversight	All human sperm samples were processed in accordance to ReproMed Ireland's standard procedures and ethical approval was attained from the University College Dublin Human Ethics Committee (protocol number LS-16-53-ODoherty-Fair).

Note that full information on the approval of the study protocol must also be provided in the manuscript.

ChIP-seq

Data deposition

- [ ] Confirm that both raw and final processed data have been deposited in a public database such as GEO.
- [ ] Confirm that you have deposited or provided access to graph files (e.g. BED files) for the called peaks.

Data access links *May remain private before publication.*	We will deposit our data to GEO and inform accession number, or provide the private link as soon as possible.
Files in database submission	We will provide all list of files after submission to GEO.
Genome browser session (e.g. UCSC)	Not available.

Methodology

Replicates	Xenopus sperm ICeChIP-seq, egg extract treated Xenopus sperm ChIP-seq, St7 Embryo ChIP-seq.were performed for 2-3 replicates per antibody. Human sperm ICeChIP-seq was performed from once.
Sequencing depth	The total number of reads for each experiment is between 15M~50M, and the samples were sequenced using Illumina HiSeq 1500 with paired-end sequenced.
Antibodies	Xenopus sperm, Xenopus egg extract treated sperm, human spermHistone H3K4me3 antibody (abcam, ab8580): This antibody can be used ChIP, immunoprecipitation, Flow cytometry, Western Blotting and Immunofluorescence in Mouse, Rat,

Rabbit, Human, Pig, Saccharomyces cerevisiae, Tetrahymena, Xenopus laevis, Arabidopsis thaliana, Caenorhabditis elegans, Drosophila melanogaster, Zebrafish, Trypanosoma cruzi, Common marmoset, Rice, Xenopus tropicalis according to the manufacture's description. There are validation data for ChIP with U-2 OS cells on the manufacture's website. In our experiment, we validated this antibody by performing ChIP-seq with Xenopus sperm, Xenopus egg extract treated sperm, human sperm, and Xenopus St7 embryos. and WB with Xenopus sperm. We use 2 ug antibody for each ChIP reaction.

Histone H3K27me3 antibody (generous gift from Dr. Thomas Jenuwein): This antibody can be used ChIP, Western Blotting in Mouse, Xenopus laevis according to his group (Peters et al., 2003) and our previous results (Teperek et al., 2016). In our experiment, we validated this antibody by performing ChIP-seq with Xenopus sperm, Xenopus egg extract treated sperm, and human sperm. We use 2 ul antibody for each ChIP reaction.

Peak calling parameters

Peaks have been called using MACS2 (v2.1.1, -q 0.01) using default options for H3K4me3 and broad option for H3K27me3.

Data quality

Adapters have been trimmed using cutadapt (v1.0). Resulting reads have been aligned to the genome by BWA (v0.6.2) mem with default options and excluding multiple alignments. The genome used for the alignment is Xlaevis 6.1 for frog and hg38 for human. In the case of ICeChIP-seq, a pseudo-genome has been assembled by including the sequence of the synthetic nucleosome ladder. Whenever it was the case, if the same sample was re-sequenced more than one time, bams file with the aligned reads have been merged before identifying duplicates.
Duplicates have been marked with Picard (v2.14 - MarkDuplicates) and removed and reads with quality below 20 were also excluded (samtools view –q 20) with samtools (v0.1.8). BAM files from ICeChIP samples have been sorted, unmapped reads and secondary alignment removed (samtools fixmate). Properly paired reads were joined to obtain fragments size information and stored in BEDPE file format with bedtools (v2.25.0). We obtained ~7x genome coverage. Peaks have been called using MACS2 (v2.1.1, -q 0.01) using default options for H3K4me3 and broad option for H3K27me3.
For analysis of ICeChIP-seq data, as indicated in the original work, the coverage of each member of the ladder in both pull down and input was used to estimate the correction factor for each mark. This factor was then used to estimate HMD either genome wide, on the genome binned in 50bp-windows or at detected peaks, as described before (Grzybowski et al., 2015).
For analysis of input samples for identification of regions enriched for different particle types, we built a R (v3.2.4) function that instructs a Hidden markov Model (HMM) and used it as an unsupervised classificatory system. For analysis of published RNA-seq data (GSE73430), we performed adapter trimming (cutadapts, options -q 10 -O 3 –m10). paired-end reads have been aligned to genome version Xlaevis 6.1 using tophat2 (options: -g 1 -p 8 --library type fr-unstranded).
In human sperm ICeChIP-seq analysis, the peaks identified are well conserved between our study and previous work (Hammoud et al., 2009). These numbers are shown in Table S6.

Software

Cutadapt (v1.0), BWA (v0.6.2), Picard (v2.14), Samtools (v0.1.8), Bedtools (v2.25.0), MACS2 (v2.1.1), R (v3.2.4), Tophat2, htseq-count, Edge R
