# Supplementary material for: Epigenetic homogeneity in histone methylation underlies sperm programming for embryonic transcription
Source: Nat Commun. 2020 Jul 13;11:3491. doi: 10.1038/s41467-020-17238-w (PMC7359334; doi:10.1038/s41467-020-17238-w)
Supplement: Supplementary file 14 — Source Data [file 41467_2020_17238_MOESM14_ESM.zip › source data/Supplementary Figure 4E digested DNA.pdf]

Filename: 2015-07-01-01 sperm progeni egg extract MNase.D1000

Gel Images

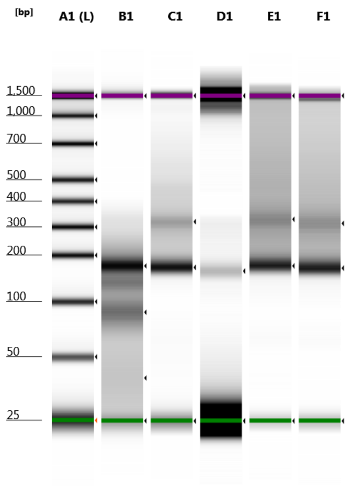

Default image (Contrast 50%), Image is Scaled to Sample, Image is Scaled to view larger Molecular Weight range

Sample Info

Well	Conc. [ng/µl]	Sample Description	Alert	Observations
A1	21.9	Ladder		Ladder
B1	53.0	Sperm		
C1	22.1	Sperm egg extract Gemi +		
D1	0.232	Sperm egg extract Gemi -		
E1	24.0	Progeni egg extract Gemi +		
F1	29.9	Progeni egg extract Gemi -		

 © Agilent Technologies, Inc. 2014
