# Supplementary material for: Epigenetic homogeneity in histone methylation underlies sperm programming for embryonic transcription
Source: Nat Commun. 2020 Jul 13;11:3491. doi: 10.1038/s41467-020-17238-w (PMC7359334; doi:10.1038/s41467-020-17238-w)
Supplement: Supplementary file 14 — Source Data [file 41467_2020_17238_MOESM14_ESM.zip › source data/Figure 1D digested DNA_sucrose.pdf]

Filename: 2016-06-03-01 Sucrose 5-15% -BSA.HSD1000

Gel Images

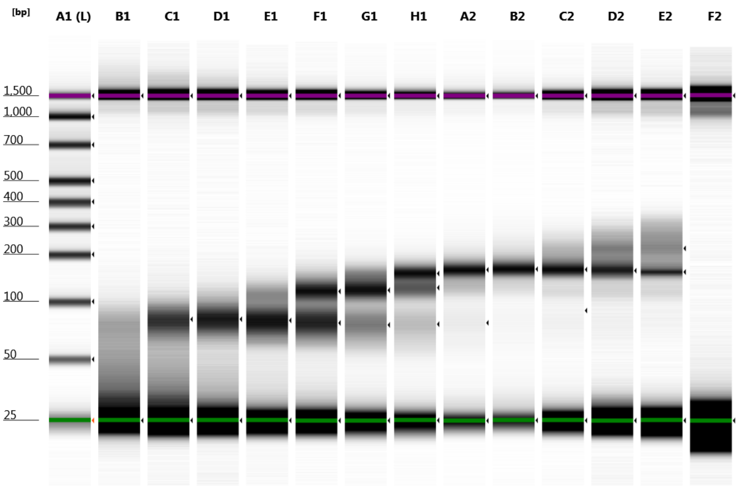

Default image (Contrast 50%), Image is Scaled to Sample, Image is Scaled to view larger Molecular Weight range

Sample Info

Well	Conc. [pg/µl]	Sample Description	Alert	Observations
A1	2340	Ladder		Ladder
B1		5-15% F13		
C1	144	5-15% F15		
D1	165	5-15% F17		
E1	125	5-15% F19		
F1	410	5-15% F21		
G1	544	5-15% F23		
H1	624	5-15% F25		
A2	416	5-15% F27		
B2	411	5-15% F29		
C2	187	5-15% F33		
D2	85.5	5-15% F35		
E2	106	5-15% F37		
F2		empty		
