# Supplementary material for: Epigenetic homogeneity in histone methylation underlies sperm programming for embryonic transcription
Source: Nat Commun. 2020 Jul 13;11:3491. doi: 10.1038/s41467-020-17238-w (PMC7359334; doi:10.1038/s41467-020-17238-w)
Supplement: Supplementary file 14 — Source Data [file 41467_2020_17238_MOESM14_ESM.zip › source data/Supplementary Figure 8A digested DNA.pdf]

Filename: 2016-11-24-01 Alan MNase sample retapesation.D1000

Gel Images

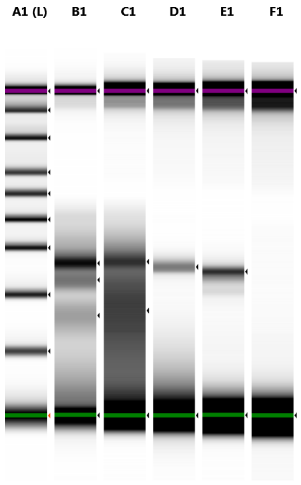

Default image (Contrast 50%), Image is Scaled to Sample, Image is Scaled to view larger Molecular Weight range

Sample Info

Well	Conc. [ng/µl]	Sample Description	Alert	Observations
A1	18.8	Ladder		Ladder
B1	11.0	Xenopus 2.5 U		
C1	4.52	Alan 0.5 U		
D1	0.502	Alan 2.5 U		
E1	0.583	Alan 15 U		
F1		empty		

© Agilent Technologies, Inc. 2016
