# Supplementary figures and images for: Epigenetic homogeneity in histone methylation underlies sperm programming for embryonic transcription
Source: Nat Commun. 2020 Jul 13;11:3491. doi: 10.1038/s41467-020-17238-w (PMC7359334; doi:10.1038/s41467-020-17238-w)

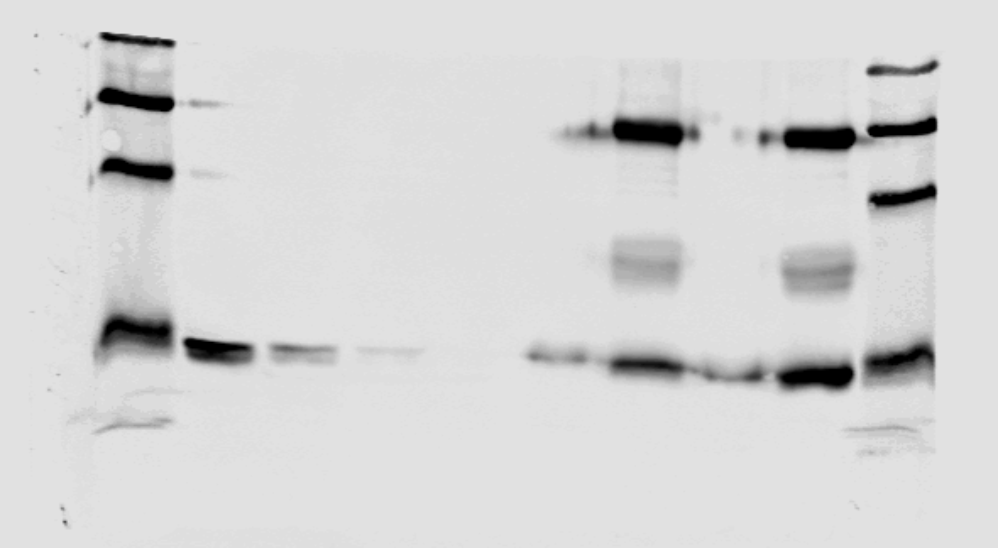

Supplement: Supplementary file 14 — Source Data [file 41467_2020_17238_MOESM14_ESM.zip › source data/Supplementary Figure 3C H3K4me3.tif]

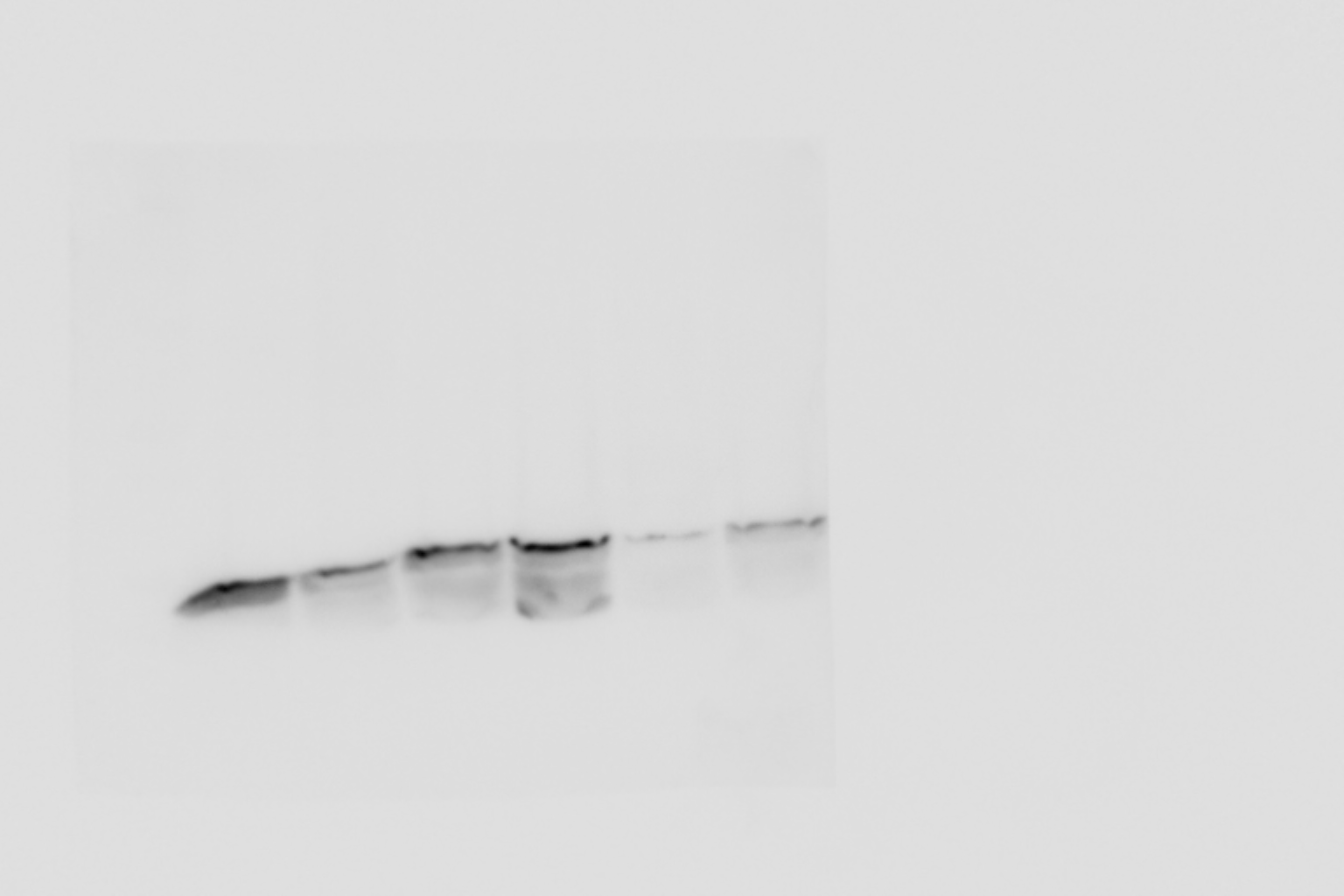

Supplement: Supplementary file 14 — Source Data [file 41467_2020_17238_MOESM14_ESM.zip › source data/Suppl Figure 2G R1_H3K4me3.tif]

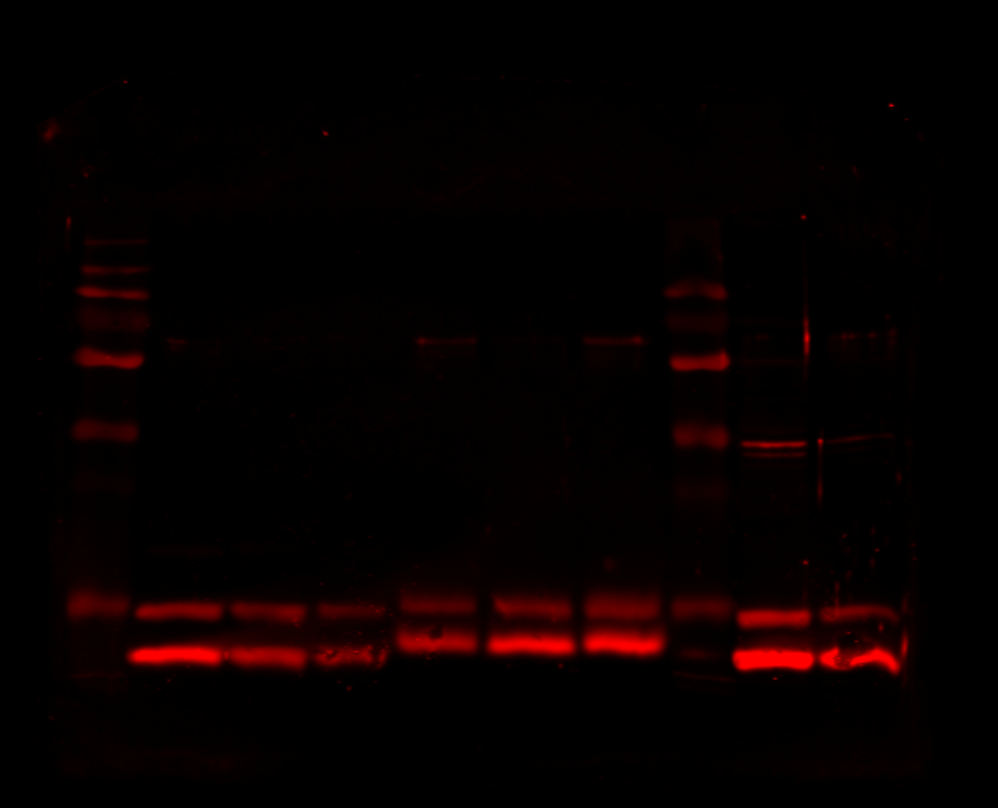

Supplement: Supplementary file 14 — Source Data [file 41467_2020_17238_MOESM14_ESM.zip › source data/Figure 1E H3 and H4.tif]

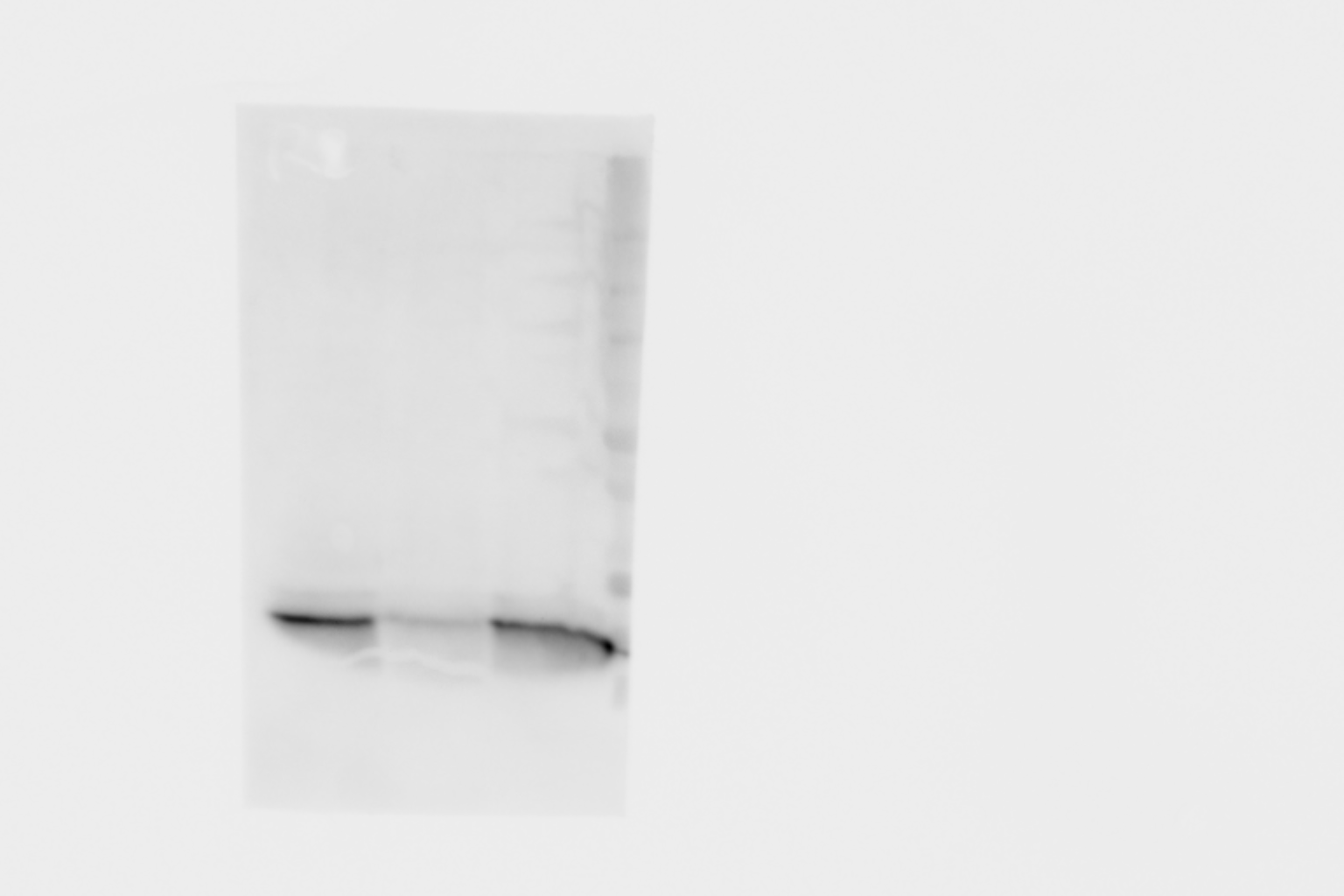

Supplement: Supplementary file 14 — Source Data [file 41467_2020_17238_MOESM14_ESM.zip › source data/Suppl Figure 2G R2_H4.tif]

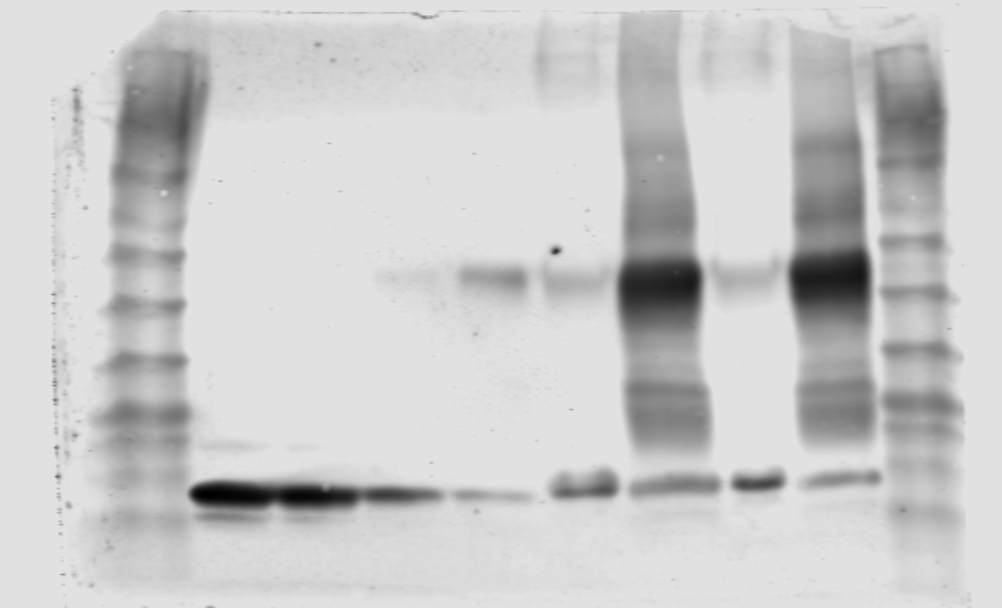

Supplement: Supplementary file 14 — Source Data [file 41467_2020_17238_MOESM14_ESM.zip › source data/Supplementary Figure 3D H3K27me3.tif]

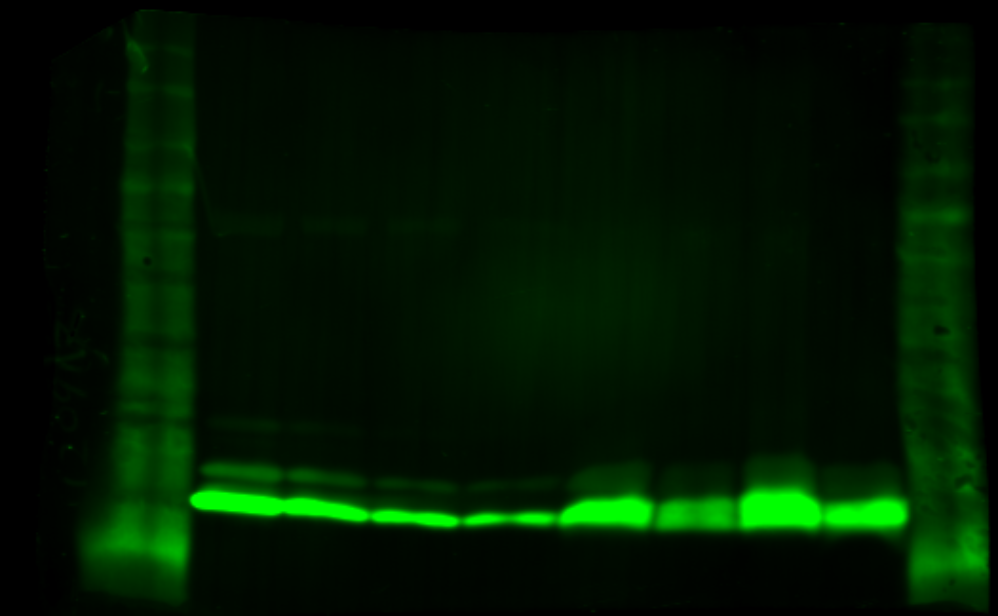

Supplement: Supplementary file 14 — Source Data [file 41467_2020_17238_MOESM14_ESM.zip › source data/Supplementary Figure 1B H4.tif]

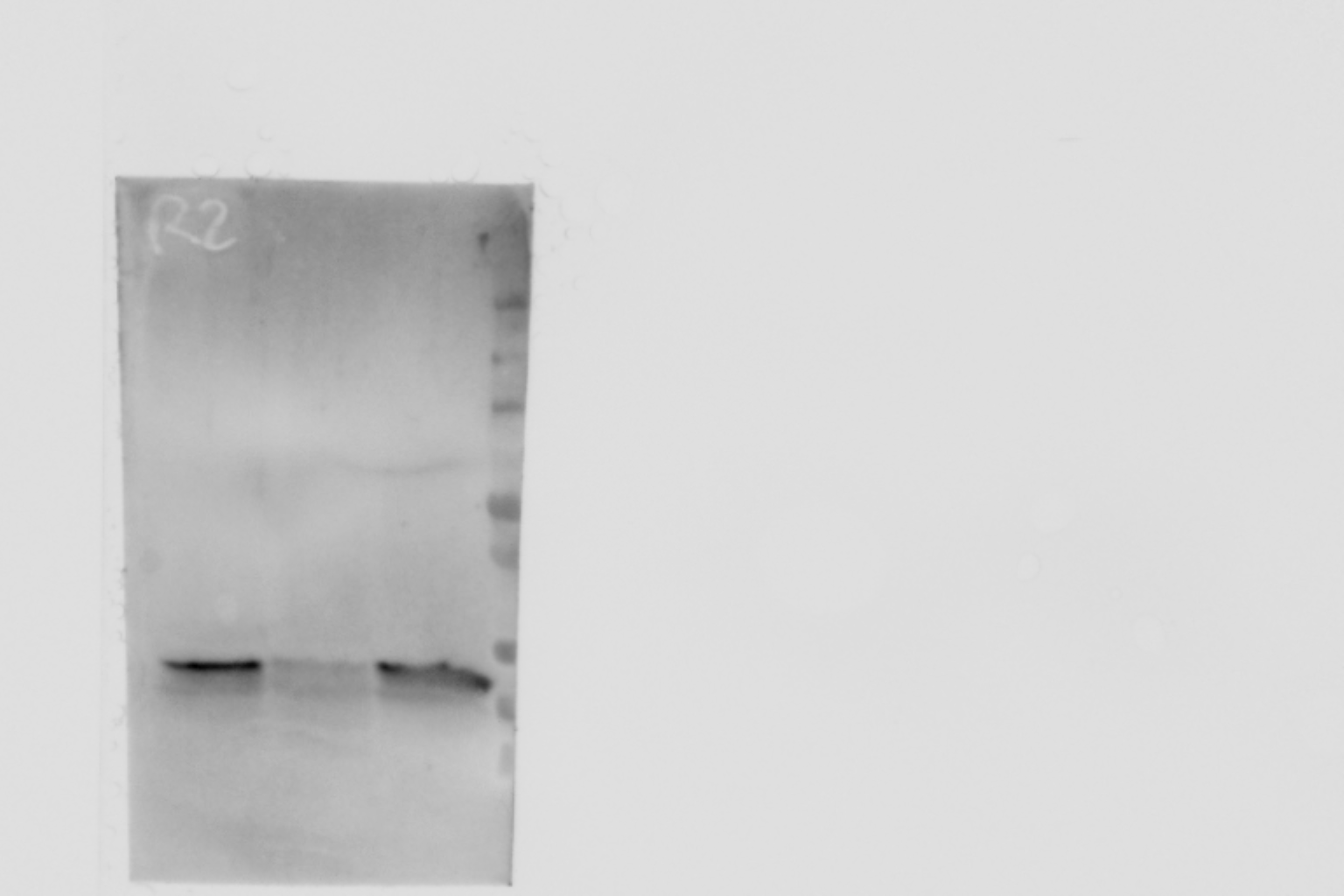

Supplement: Supplementary file 14 — Source Data [file 41467_2020_17238_MOESM14_ESM.zip › source data/Suppl Figure 2G R2_H3K27me3.tif]

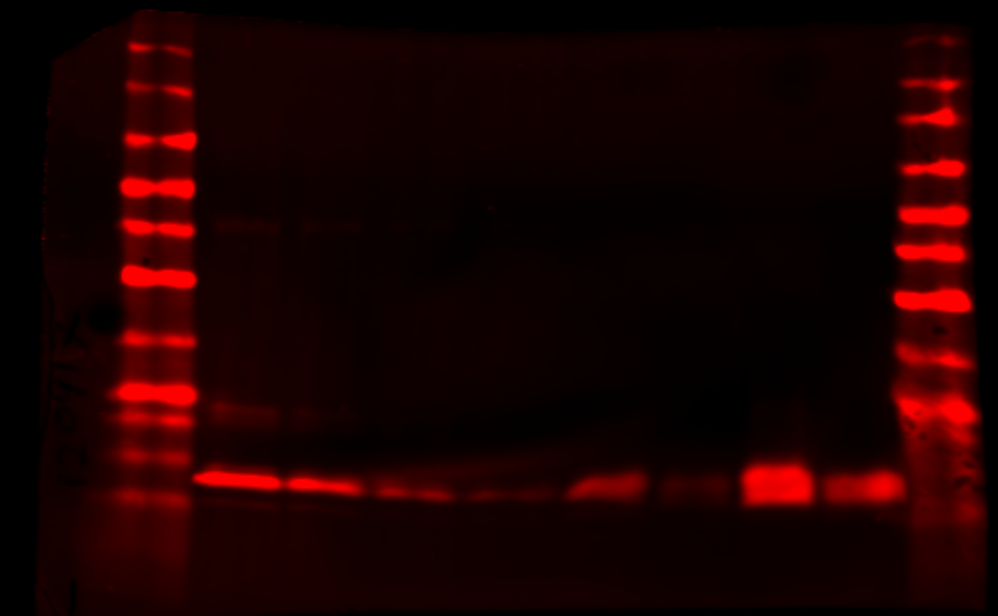

Supplement: Supplementary file 14 — Source Data [file 41467_2020_17238_MOESM14_ESM.zip › source data/Supplementary Figure 1B H2A.tif]

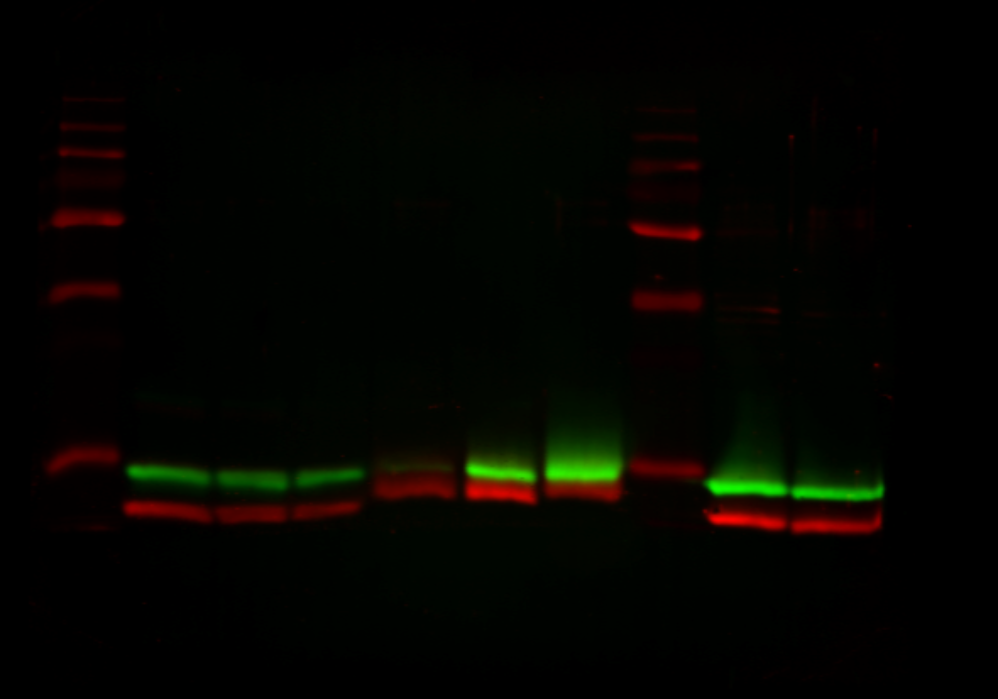

Supplement: Supplementary file 14 — Source Data [file 41467_2020_17238_MOESM14_ESM.zip › source data/Figure 1E down H2B and H4.tif]

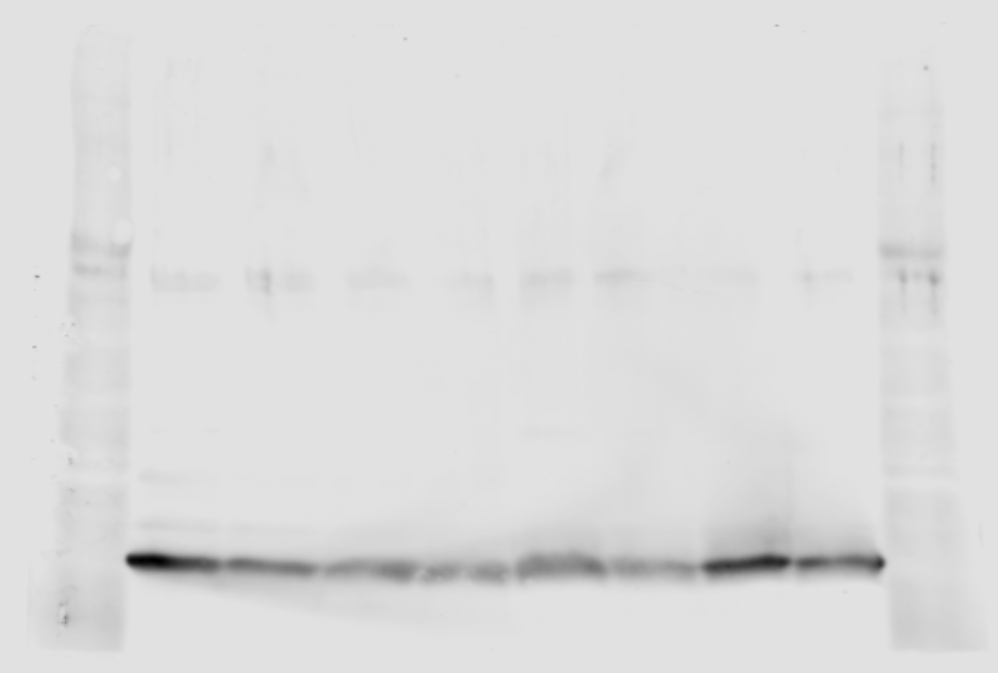

Supplement: Supplementary file 14 — Source Data [file 41467_2020_17238_MOESM14_ESM.zip › source data/Supplementary Figure 1A H4.tiff]

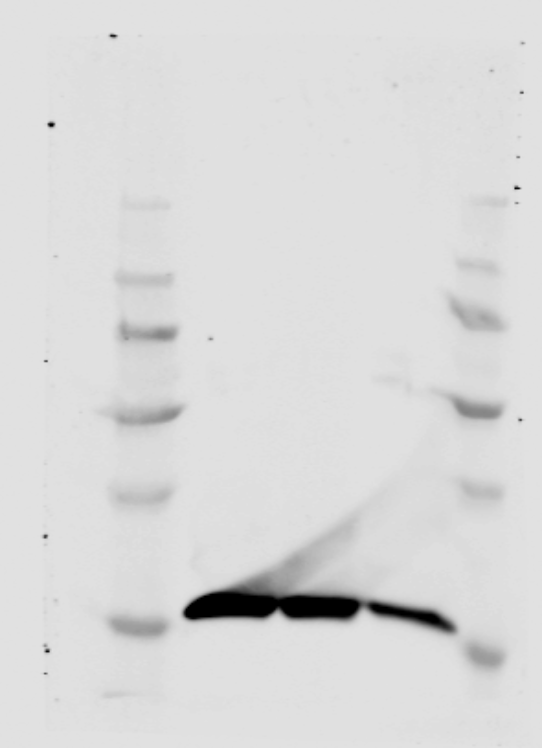

Supplement: Supplementary file 14 — Source Data [file 41467_2020_17238_MOESM14_ESM.zip › source data/Supplementary Figure 2C H3.tif]

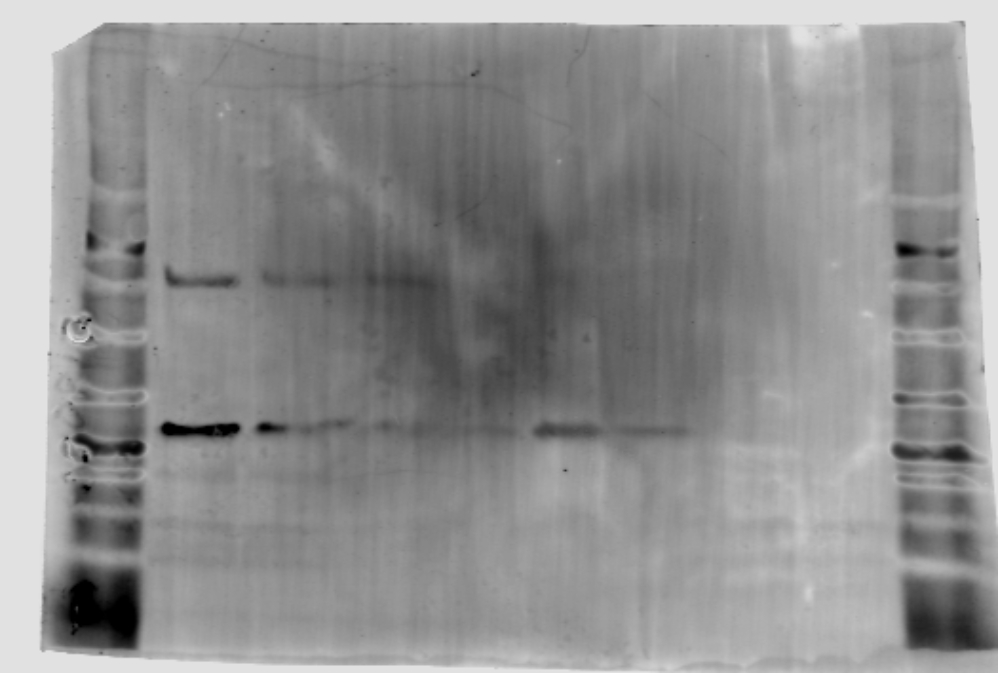

Supplement: Supplementary file 14 — Source Data [file 41467_2020_17238_MOESM14_ESM.zip › source data/Supplementary Figure 1A HMGB1.tiff]

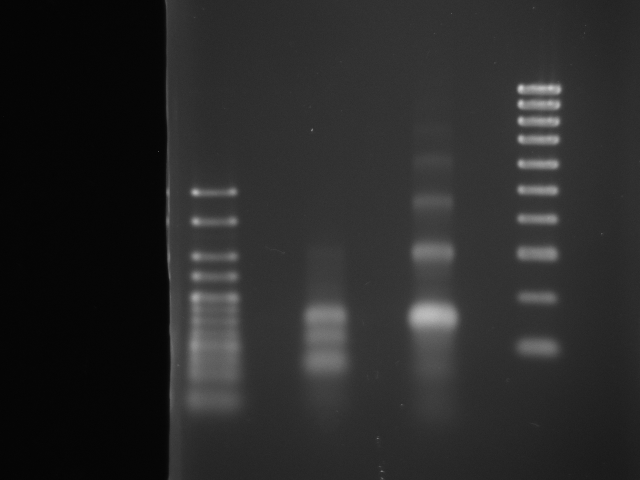

Supplement: Supplementary file 14 — Source Data [file 41467_2020_17238_MOESM14_ESM.zip › source data/Figure 1B digested DNA.tif]

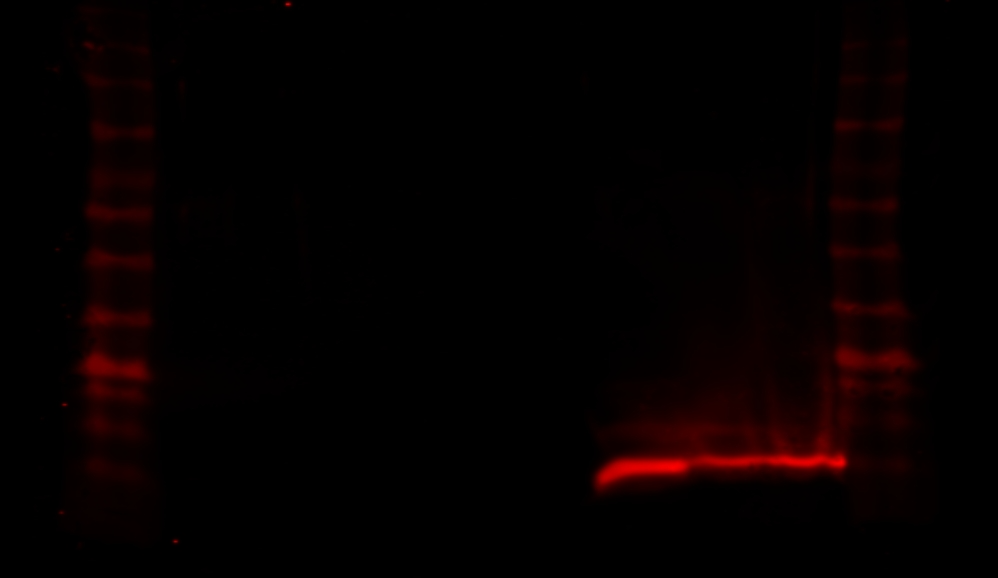

Supplement: Supplementary file 14 — Source Data [file 41467_2020_17238_MOESM14_ESM.zip › source data/Supplementary Figure 1C H4.tiff]

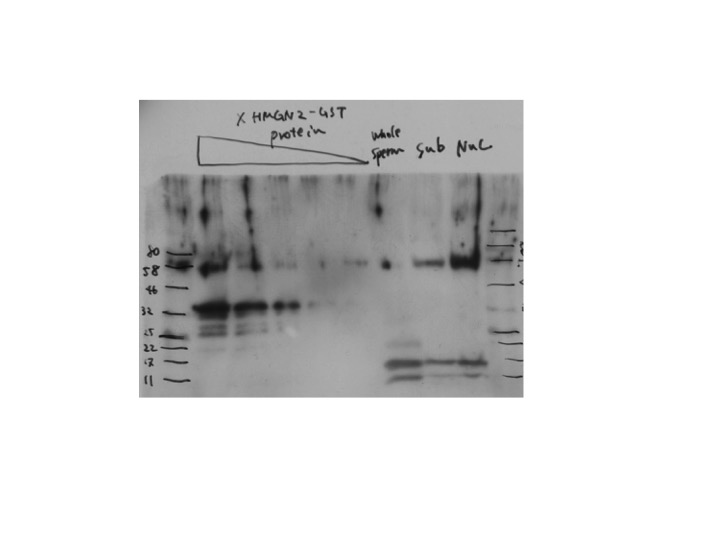

Supplement: Supplementary file 14 — Source Data [file 41467_2020_17238_MOESM14_ESM.zip › source data/Supplementary Figure 1C HMGN2.tiff]
